# Projected Life Expectancy for Adolescents With HIV in the US

**DOI:** 10.1001/jamahealthforum.2024.0816

**Published:** 2024-05-10

**Authors:** Anne M. Neilan, Ogochukwu L. Ufio, Isaac Ravi Brenner, Clare F. Flanagan, Fatma M. Shebl, Emily P. Hyle, Kenneth A. Freedberg, Andrea L. Ciaranello, Kunjal Patel

**Affiliations:** 1 Division of General Academic Pediatrics, Department of Pediatrics, Massachusetts General Hospital, Boston; 2 Medical Practice Evaluation Center, Massachusetts General Hospital, Boston; 3 Division of Infectious Diseases, Department of Medicine, Massachusetts General Hospital, Boston; 4 Harvard Medical School, Boston, Massachusetts; 5 Harvard University Center for AIDS Research, Cambridge, Massachusetts; 6 Division of General Internal Medicine, Massachusetts General Hospital, Boston; 7 Department of Epidemiology, Center for Biostatistics in AIDS Research, Harvard T.H. Chan School of Public Health, Boston, Massachusetts

## Abstract

**Importance:**

Life expectancy is a key measure of overall population health. Life expectancy estimates for youth with HIV in the US are needed in the current HIV care and treatment context to guide health policies and resource allocation.

**Objective:**

To compare life expectancy between 18-year-old youth with perinatally acquired HIV (PHIV), youth with nonperinatally acquired HIV (NPHIV), and youth without HIV.

**Design, Setting, and Participants:**

Using a US-focused adolescent-specific Monte Carlo state-transition HIV model, we simulated individuals from age 18 years until death. We estimated probabilities of HIV treatment and care engagement, HIV progression, clinical events, and mortality from observational cohorts and clinical trials for model input parameters. The simulated individuals were 18-year-old race and ethnicity–matched youth with PHIV, youth with NPHIV, and youth without HIV; 47%, 85%, and 50% were assigned male sex at birth, respectively. Individuals were categorized by US Centers for Disease Control and Prevention–defined HIV acquisition risk: men who have sex with men, people who ever injected drugs, heterosexually active individuals at increased risk for HIV infection, or average risk for HIV infection. Distributions were 3%, 2%, 12%, and 83% for youth with PHIV and youth without HIV, and 80%, 6%, 14%, and 0% for youth with NPHIV, respectively. Among the simulated youth in this analysis, individuals were 61% Black, 24% Hispanic, and 15% White, respectively.

**Exposures:**

HIV status by timing of acquisition.

**Main Outcomes:**

Life expectancy loss for youth with PHIV and youth with NPHIV: difference between mean projected life expectancy under current and ideal HIV care scenarios compared with youth without HIV. Uncertainty intervals reflect varying adolescent HIV-related mortality inputs (95% CIs).

**Results:**

Compared with youth without HIV (life expectancy: male, 76.3 years; female, 81.7 years), male youth with PHIV and youth with NPHIV had projected life expectancy losses of 10.4 years (95% CI, 5.5-18.1) and 15.0 years (95% CI, 9.3-26.8); female youth with PHIV and youth with NPHIV had projected life expectancy losses of 11.8 years (95% CI, 6.4-20.2) and 19.5 years (95% CI, 13.8-31.6), respectively. When receiving ideal HIV care, life expectancy losses were projected to improve for youth with PHIV (male: 0.5 years [95% CI, 0.3-1.8]: female: 0.6 years [95% CI, 0.4-2.1]) but were projected to persist for youth with NPHIV (male: 6.0 years [95% CI, 5.0-9.1]; female: 10.4 years [95% CI, 9.4-13.6]).

**Conclusions:**

This adolescent-focused microsimulation modeling analysis projected that youth with HIV would have shorter life expectancy than youth without HIV. Projected differences were larger for youth with NPHIV compared with youth with PHIV. Differences in mortality by sex at birth, sexual behavior, and injection drug use contributed to lower projected life expectancy among youth with NPHIV. Interventions focused on HIV care and social factors are needed to improve life expectancy for youth with HIV in the US.

## Key Points

**Question:**

How does life expectancy compare for 18-year-old youth who acquired HIV perinatally (PHIV), youth who acquired HIV nonperinatally (NPHIV), and youth without HIV in the US?

**Findings:**

In an adolescent-specific microsimulation model, youth with PHIV and youth with NPHIV had lower life expectancy than youth without HIV; youth with NPHIV had lower life expectancy compared with youth with PHIV. The life expectancy gap between youth with PHIV and youth without HIV decreased in an ideal HIV care scenario, but persisted for youth with NPHIV.

**Meaning:**

The study results suggest that interventions focused on HIV care and the social factors associated with disparities in non–HIV-related mortality are needed to improve life expectancy for youth with HIV in the US.

## Teaser

In this modeled study, life expectancy was compared among 18-year-old youth with perinatally acquired HIV, youth with nonperinatally acquired HIV, and youth without HIV.

## Introduction

Approximately 45 900 youths ages 13 to 24 years in the US are living with HIV^1^; they include those who acquired HIV perinatally, either in utero or around the time of birth (youth with PHIV; 15% of youth with HIV) and those who acquired HIV nonperinatally or later in life (youth with NPHIV; 85% of youth with HIV).^2^ These 2 groups differ in important ways. Youth with PHIV who matured into adolescence with HIV primarily initiated treatment with suboptimal antiretroviral therapy (ART) and formulations in infancy and many have developed resistant variants over time.^3,4^ Many youth with PHIV receive complicated ART regimens and may experience poorer virologic responses when switching to receiving integrase strand transfer inhibitor (INSTI)–based regimens compared with youth with NPHIV.^3,5^ Youth with PHIV also face challenges transitioning from dependence on adult caregivers to self-care and may have an increased risk of long-term complications of HIV and ART.^3^ Youth with NPHIV are more likely to be male at birth, men who have sex with men (MSM), people who ever injected drugs (WID), or heterosexually active individuals with sexual contacts placing them at increased risk for HIV infection.^6^ They are more likely to have a new diagnosis and have more recently started to receive HIV care.^6^ Youth with NPHIV receiving care are less likely to be prescribed ART compared with youth with PHIV.^6^

Life expectancy is a population health indicator that can direct health policies and resource allocation.^7^ Improvements in HIV care have been associated with increased life expectancy for adults with HIV, with the projected life expectancy gap between adults living with and without HIV in the US now ranging from 8 to 13 years.^8,9,10^ Whether these benefits apply to or differ for youth with PHIV and youth with NPHIV in the US is unknown. Additionally, youth with PHIV who are aged 18 years are the first generation of young adults who acquired HIV in infancy, and long-term data on their survival are lacking; projected estimates of their life expectancy as they age into adulthood are needed to inform long-term HIV care.

We developed an adolescent-specific microsimulation model to project and compare life expectancy between 18-year-old youth with PHIV, youth with NPHIV, and youth without HIV in the US. The model specifically accounts for age and cause-specific mortality due to HIV and non–HIV-related causes that occur during the substantial time youth with HIV spend out of care, which few other studies consider.^11^ We additionally projected life expectancy within a scenario in which HIV care was optimized to support maintenance of high CD4 counts, ART treatment adherence, and retention in care.

## Methods

### Analytical Overview

We developed the Cost-Effectiveness of Preventing AIDS Complications (CEPAC)–Adolescent model^12,13^ to project life expectancy for 18-year-olds with and without HIV in the US. We simulated 3 age-matched, race-matched, and ethnicity-matched cohorts based on available national data reporting similar race and ethnicity distributions among youth with HIV: youth with PHIV, youth with NPHIV, and youth without HIV.^2,6,14,15^ Characteristics of youth with PHIV and youth with NPHIV, including initial CD4 counts, treatment histories, adherence to ART and care, and rates of opportunistic infections and deaths, were informed by cohort and clinical trial data from the Adolescent Medicine Trials Network for HIV/AIDS Interventions (ATN), International Maternal Pediatric Adolescent AIDS Clinical Trials (IMPAACT) Network, and published data. In the model, youth with PHIV and youth with NPHIV cohorts experience cause-specific HIV-related mortality, and all cohorts experience non–HIV-related mortality as stratified by sex at birth, age, race and ethnicity, and HIV acquisition risk. HIV acquisition risk categories were based on US Centers for Disease Control and Prevention (CDC) definitions: MSM, PWID, and heterosexually active individuals at increased risk for HIV infection.^16^ We projected survival curves and life expectancy from age 18 years for each cohort by sex at birth and risk for HIV acquisition, including the range of model results when varying adolescent HIV-related mortality inputs through their 95% CIs, which are the most uncertain parameters.

Research projects using the CEPAC model were approved by the Mass General Brigham human research committee. We used the Consolidated Health Economic Evaluation Reporting Standards (CHEERS) reporting guidelines. The need for informed consent was waived in this study, as no individual-level data were used.

### Model Structure

The CEPAC-Adolescent model is a Monte Carlo state-transition microsimulation of HIV disease and treatment.^12,13^ The model tallies monthly clinical events for each individual from model entry until death.

#### HIV Disease Progression, Treatment, and Care

Youth with PHIV and youth with NPHIV cohorts enter the model as already having a diagnosis of HIV, having been prescribed INSTI-based ART, and receiving care. When initiating treatment with an ART regimen, youth experience a probability of achieving virologic suppression depending on a baseline adherence level. Those achieving virologic suppression experience a rise in CD4 counts. They also face a monthly probability of subsequently developing viremia, along with probabilities of either resuppressing on their initial regimen or switching regimens. Youth with HIV also face probabilities of loss to follow-up and return to HIV care. Youth without HIV never acquire HIV in their lifetimes for comparison.

#### Modeled Mortality

All modeled cohorts face risks of non–HIV-related mortality depending on age, sex at birth, race and ethnicity, and HIV acquisition risk group. Youth with HIV face additional age-stratified and CD4-stratified risks of HIV-related mortality.

### Model Inputs

#### Modeled Cohorts

All cohorts enter the model at the same age (18 years) and with the same race and ethnicity distribution: 61% Black, 24% Hispanic, and 15% White, as based on CDC-reported race and ethnicity distributions for youth with PHIV and youth with NPHIV (Table^2,13,14,15,17,18,19,20,21,22,23,24,25,26,27,28,29,30,31,32,33,34,35,36,37,38,39^; eTable 1 in Supplement 1).^2^ Among the youth with PHIV cohort, 47% are assigned male sex at birth compared with 85% of youth with NPHIV and 50% of youth without HIV, corresponding to the observed distribution of sex at birth in these populations.^2^

**Table.** Input Parameters for a Simulation Model to Estimate Life Expectancy Among Youth With HIV in the US

Base-case parameter	Youth with PHIV	Youth with NPHIV	Youth without HIV	Source
Age, y	18	18	18	Modeled population
Black/Hispanic/White, %	61/24/15	61/24/15	61/24/15	US Centers for Disease Control and Prevention^2^, Neilan et al^14^
Female/male sex at birth, %	53/47	15/85	50/50	US Centers for Disease Control and Prevention^2^
Distribution of risk factors for HIV acquisition^a^				
Male/female, %				
Men who have sex with men	6/0	93/0	6/0	US Centers for Disease Control and Prevention^2^
People who ever injected drugs	3/2	4/10	3/2	
Heterosexually active individual at increased risk for HIV infection	14/9	3/90	14/9	
Average risk for HIV infection	77/89	0/0	77/89	
**Characteristic**	**Youth with PHIV**	**Youth with NPHIV**		
Baseline HIV-related characteristics				
CD4 count at model start, mean (SD), cells/μL	635 (381)	527 (227)		Neilan et al^15^
HIV RNA set point while not receiving ART				
Mean log_10_ copies/mL	5.22 (165 800)	5.22 (165 800)		Daar et al^17^
Baseline ART adherence and virologic suppression				
Adherence to ART, % of initial cohort				
Adherence >90%	40	40		Neilan et al^13^
Adherence <90%	60	60		
ART efficacy, %^b^				
INSTI-based regimen^c^				
>95% Adherence	80.0	96.4		Briand et al^18^, Walmsley et al^19^, Gaur et al^20^
PI-based regimen^d^				
>95% Adherence	NA	88.0		Cahn et al^21^
NNRTI-based regimen				
>95% Adherence	93.0	93.0		Turkova et al^22^
Salvage regimen				
>95% Adherence	88.0	88.0		Clotet et al^23^
Virologic failure by ART regimen, monthly probability	0.002-0.180	0.002-0.180		Cheng et al^24^, Gachara et al,^25^ Wu et al^26^
Loss to follow-up				
Loss to follow-up after 12 mo, range by adherence level, monthly probability	0.068-0.0001	0.068-0.0001		Agwu et al^27^, Fleishman et al^28^
Returning to care, monthly probability	0.015	0.015		Helleberg et al^29^
WHO stage 4 opportunistic infections while not receiving ART, range by CD4 count, monthly probability^e^				
Age 18-30 y	0.0008-0.0087	0.0000-0.0340		Neilan et al^15^
Age ≥30 y	0.0005-0.0312	0.0005-0.0312		Multicenter AIDS Cohort Study Public Dataset^30^
Adolescent HIV-related mortality, monthly probability, by CD4 count, cells/μL				
>500	0.00002-0.00006	0.00013-0.00067		Neilan et al^15^
351-500	0.00031-0.00094	0.00040-0.00262		
201-350	0.00031-0.00094	0.00040-0.00262		
101-200	0.00256-0.00575	0.00059-0.00719		
51-100	0.00256-0.00575	0.00059-0.00719		
0-50	0.00256-0.00575	0.00059-0.00719		
Adult HIV-related mortality				
Death of WHO Stage 4 opportunistic infection, monthly probability	0.03741	0.03741		Multicenter AIDS Cohort Study Public Dataset^30^
Chronic AIDS death, monthly probability, by CD4 count, cells/μL				Multicenter AIDS Cohort Study Public Dataset^30^, Ray et al,^31^ Engels et al,^32^ Edwards et al^33^
>500	0.00002-0.00005	0.00002-0.00005		
351-500	0.00002-0.00015	0.00002-0.00015		
201-350	0.00005-0.00031	0.00005-0.00031		
101-200	0.00003-0.00394	0.00003-0.00394		
51-100	0.00002-0.00388	0.00002-0.00388		
0-50	0.00009-0.00824	0.00009-0.00824		
Non–HIV-related mortality, by age, monthly probability, y				
Female individuals				US Centers of Disease Control and Prevention^2^, National Bureau of Economic Research^34^, Human Mortality Database^35^
18-19	0.00005-0.00006	0.00016-0.00018		
20-24	0.00006-0.00007	0.00018-0.00022		
25-29	0.00008-0.00010	0.00024-0.00030		
30-39	0.00011-0.00017	0.00032-0.00050		
40-49	0.00018-0.00027	0.00033-0.00080		
50-59	0.00026-0.00052	0.00047-0.00094		
60-69	0.00054-0.00115	0.00099-0.00212		
70-79	0.00122-0.00293	0.00226-0.00546		
80-100	0.00323-1.00000	0.00603-1.00000		
Male individuals				
18-19	0.00011-0.00013	0.00020-0.00024		
20-24	0.00014-0.00018	0.00026-0.00033		
25-29	0.00018-0.00020	0.00033-0.00038		
30-39	0.00019-0.00026	0.00036-0.00049		
40-49	0.00027-0.00040	0.00037-0.00069		
50-59	0.00043-0.00093	0.00053-0.00114		
60-69	0.00098-0.00185	0.00120-0.00230		
70-79	0.00193-0.00417	0.00241-0.00531		
80-100	0.00459-1.00000	0.00587-1.00000		

Abbreviations: ART, antiretroviral therapy; INSTI, integrase strand transfer inhibitor; NA, not applicable; NNRTI, non-nucleoside reverse transcriptase inhibitor; NPHIV, HIV acquired nonperinatally; PHIV, HIV acquired perinatally; PI, protease inhibitor; WHO, World Health Organization.

^a^ Although data are limited, risk factor distributions for youth with PHIV and youth without HIV are similar in the literature.^36,37,38^

^b^ Viral load of less than 50 copies/mL at 48 weeks.

^c^ Efficacy between 57% and 95% adherence is exponentially interpolated.

^d^ Youth with PHIV are not offered a PI-based regimen in the model; we assumed they had already been treated with a PI-based regimen earlier in life. Salvage regimens may include a PI.

^e^ Multiplier of 0.2 is applied for patients receiving ART.^39^

#### HIV Disease Progression and Treatment

At the start of the model, youth with PHIV and youth with NPHIV were assigned mean (SD) CD4 counts of 635 (381) and 527 (227) cells/μL, respectively.^15^ Youth with PHIV and youth with NPHIV are modeled with the same ART and care adherence distribution at model start and over time (mean adherence <26 years: 82%^13^; mean adherence ≥26 years: 90%^40^) because of limited data showing differences between groups (Table; eTable 2 in Supplement 1). The likelihood of virologic suppression on ART differs based on youth with PHIV-specific and youth with NPHIV-specific cohort and trial data,^18,19,20^ with an 80.0% and 96.4% chance of suppression for those with greater than 95% adherence to treatment with INSTI-based ART, respectively, likely reflecting the more extensive treatment history for youth with PHIV. Those who experience virologic failure while receiving the initial regimen may experience resuppression with the same regimen; if there is continued virologic failure, all youth with HIV may switch regimens, although youth with PHIV have fewer alternatives due to previous ART exposure.

#### Modeled Mortality

##### HIV-Related Mortality

ATN and IMPAACT data were used to derive age-stratified and CD4-stratified HIV-related mortality rates for individuals aged 18 to younger than 30 years (adolescent HIV-related mortality; eTables 3-6 in Supplement 1). Data from the Multicenter AIDS Cohort Study (participants did not receive ART),^30^ the HIV-CAUSAL Collaboration (did not receive ART),^31^ and the North American AIDS Cohort Collaboration on Research and Design (participants received ART)^32,33^ informed rates from those 30 years or older (adult HIV-related mortality; eTables 7-9 in Supplement 1).

##### Non–HIV-Related Mortality

**Figure 1.**
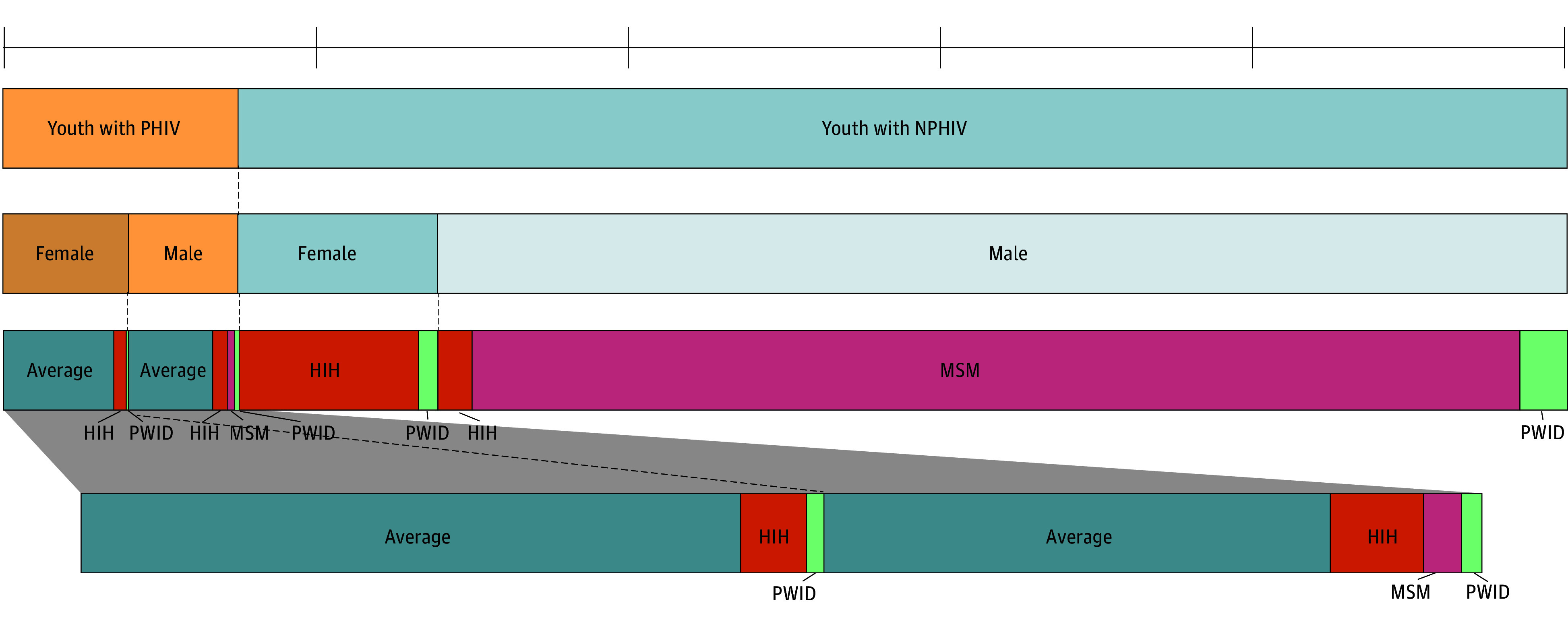
Distribution of Risk Factors for HIV Acquisition for US Youth With HIV The top bar shows the distribution of all modeled youth with HIV, broken down by youth with HIV acquired perinatally (PHIV) and youth with HIV acquired nonperinatally (NPHIV). The middle bar shows the distribution of sex assigned at birth for each of those HIV acquisition groups, with lighter shading for male individuals and darker shading for female individuals. The bottom bar shows the distribution of risk factors for HIV acquisition, as stratified by mode of HIV acquisition and sex assigned at birth. While youth with PHIV all acquired HIV perinatally, we assumed the same distribution of HIV risk factors for youth with PHIV as for the population of US youth without HIV. Average risk refers to those who do not have any identified HIV risk acquisition factors. HIH indicates heterosexually active individual at increased risk for HIV infection; MSM, men who have sex with men; PWID, people who ever injected drugs.

To account for disparities in non–HIV-related mortality in the US among HIV acquisition risk groups, the proportions of youth with PHIV, youth with NPHIV, and youth without HIV male and female cohorts, respectively, are assigned as: MSM (male: 6%, 93%, and 6%; female: not applicable), PWID (male: 3%, 4%, and 3%; female: 2%, 10%, and 2%), heterosexually active individuals at increased risk for HIV infection (male: 14%, 3%, and 14%; female: 9%, 90%, and 9%), and individuals at average risk for HIV infection (male: 77%, 0%, and 77%; female: 89%, 0%, and 89%)^2,41^ (Table and Figure 1). While youth with PHIV acquired HIV perinatally, we assumed the same distribution of HIV risk factors for youth with PHIV as the population of US youth without HIV.^36,37,38^ We assigned all youth with NPHIV to one of the HIV acquisition risk groups (ie, there were no average-risk individuals) to reflect that all individuals with HIV acquired nonperinatally are reported by the CDC in one of these categories. The National HIV Behavioral Surveillance System considers individuals who have an income level at or less than 150% of the federal poverty level to meet the definition of heterosexually active individuals at increased risk for HIV infection.^16^ We applied this precedent and used mortality data for individuals with an income level less than the federal poverty level as a proxy for mortality among heterosexually active individuals at increased risk for HIV infection (eMethods in Supplement 1).^34^ Youth with PHIV and youth without HIV not assigned to a risk group are considered at average risk for HIV infection.

Non–HIV-related mortality probabilities were derived from the Human Mortality Database (population size) and National Center for Health Statistics (causes of death; eTable 10 in Supplement 1).^34,35^ Age-stratified and sex at birth–stratified non–HIV-related mortality rates were adjusted for race and ethnicity and risk for HIV acquisition using age-stratified relative mortality ratios from the National Health and Nutrition Examination Survey (eTables 11 and 12 in Supplement 1).^41^ Because some mortality rates are significantly lower among people who survived to middle age (eg, for female PWID), we derived relative mortality ratios at less and greater than age 45 years (eTable 12 in Supplement 1).

Additionally, we modeled an ideal HIV care scenario with youth with PHIV and youth with NPHIV who enter the simulation with a CD4 count of 750 cells/μL, 99% adherence, and no loss to follow-up. To evaluate how adult HIV-related mortality and relative mortality ratios of non–HIV-related mortality contribute to the life expectancy estimates, we varied these parameters through their 95% CIs. We also varied the age threshold for non–HIV-related relative mortality ratios (eMethods and eTables 11 and 12 in Supplement 1). To evaluate the potential contribution of overestimating mortality for heterosexually active persons at increased risk for HIV by using mortality data for individuals with an income level less than the federal poverty level as a proxy for their mortality, we replaced their mortality inputs with those for individuals with an average risk for HIV infection.

## Results

### Primary Analyses: Life Expectancy Outcomes Overall and by Sex at Birth

**Figure 2.**
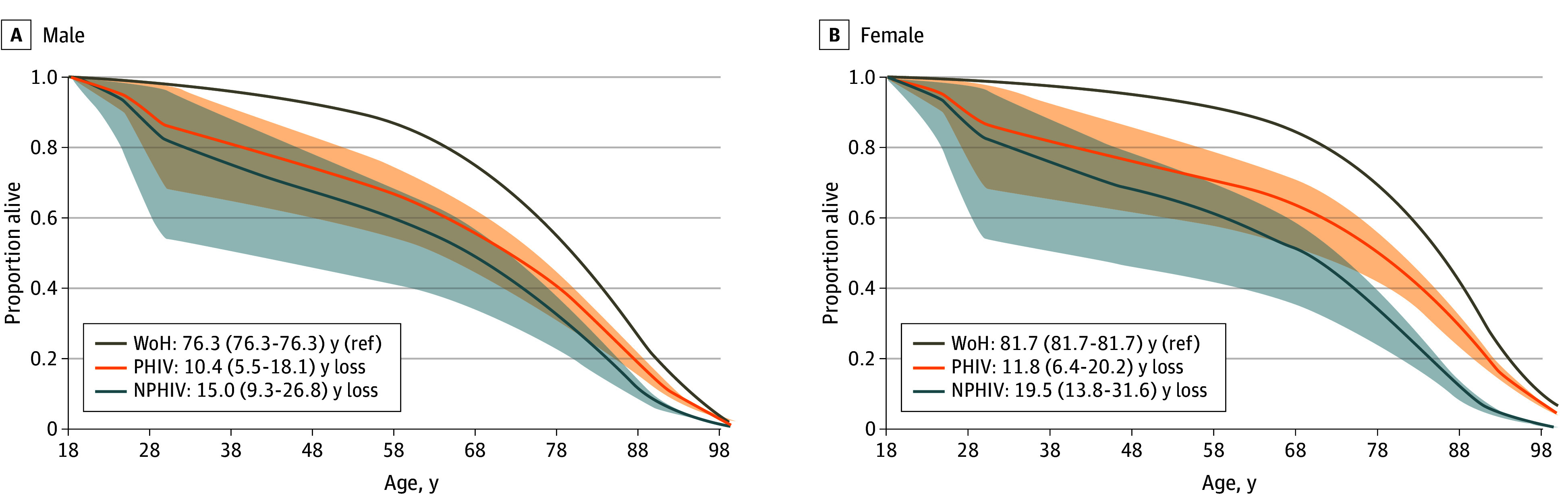
Projected Survival From Age 18 Years by Modeled Cohort The first value reported underneath the curves is total mean life expectancy for the reference group (youth without HIV [WoH]); the other numbers show life expectancy losses compared with that reference group. The shaded areas represent the range of model results when varying adolescent HIV-related mortality input data through their 95% CI. In the legend, the lower and upper estimates of life expectancy and life expectancy loss are shown in parentheses. The inflection points in the curves around age 30 years reflects the transition in the model from the adolescent HIV-related mortality inputs to the adult HIV-related mortality inputs. NPHIV indicates HIV acquired nonperinatally; PHIV, HIV acquired perinatally; ref, reference.

Overall, projected life expectancy was higher for youth without HIV (79.0 years) than youth with PHIV (68.0 years; 95% CI, 60.0-73.2) and youth with NPHIV (61.5 years. [95% CI, 49.6-67.2)]) (eTable 4 in Supplement 1). Life expectancy was 76.3 and 81.7 years for male and female youth without HIV, respectively. Compared with youth without HIV, life expectancy losses were 10.4 (95% CI, 5.5-18.1) and 15.0 (95% CI, 9.3-26.8) years for male youth with PHIV and youth with NPHIV, respectively, and 11.8 (95% CI, 6.4-20.2) and 19.5 (95% CI, 13.8-31.6) years for female youth with PHIV and youth with NPHIV, respectively (Figure 2; eTable 13 in Supplement 1).

### Life Expectancy Outcomes by Sex at Birth and Risk for HIV Acquisition

#### Male Youth

**Figure 3.**
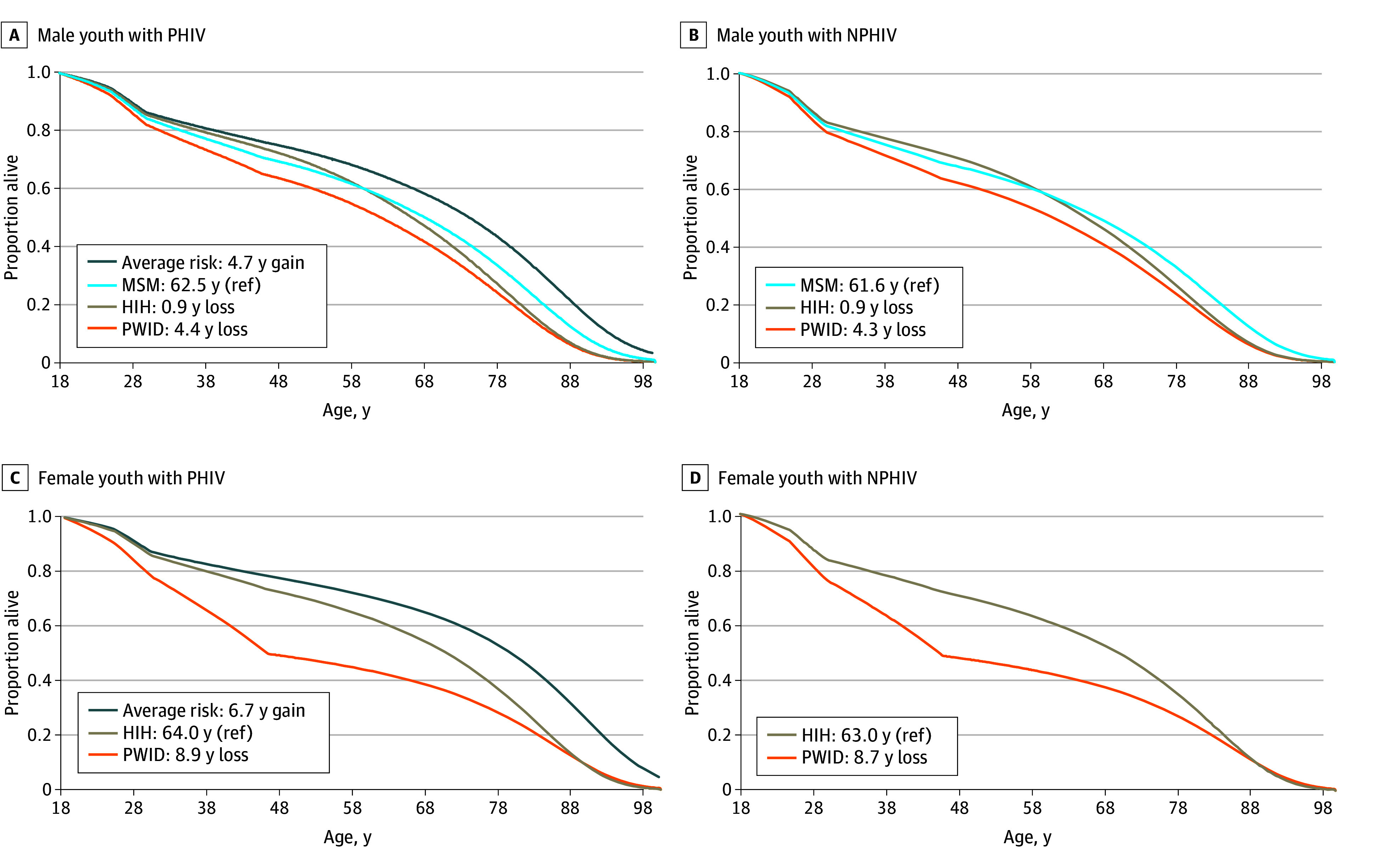
Projected Survival From Age 18 Years by Risk for HIV Acquisition The mean life expectancy for the reference cohort is indicted by the cohort denoted by (ref) in the figure; the other numbers show mean projected life expectancy gains or losses compared with that reference group. The inflection points in the female people who ever injected drugs (PWID) curves are due to the different relative mortality ratios used to adjust the non–HIV-related mortality younger than or older than age 45 years. HIH indicates heterosexually active individuals at increased risk for HIV infection; MSM, men who have sex with men; NPHIV, HIV acquired nonperinatally; PHIV, HIV acquired perinatally.

To compare life expectancy differences by risk of HIV acquisition across male youth with PHIV and youth with NPHIV cohorts, we used MSM as the reference group because they had the highest projected life expectancy among CDC-defined HIV acquisition risk groups. Male youth with PHIV who met the definition of having average risk for HIV infection were projected to have a 4.7-year life expectancy gain compared with youth with PHIV who were MSM, whereas those who met the definition of heterosexually active individuals at increased risk for HIV infection and people WID had projected life expectancy losses of 0.9 and 4.4 years, respectively (Figure 3A). Among male youth with NPHIV, heterosexually active individuals at increased risk for HIV infection and people WID had projected life expectancy losses of 0.9 and 4.3 years, respectively, compared with MSM (Figure 3B).

#### Female Youth

To compare life expectancy differences by risk of HIV acquisition across female youth with PHIV and youth with NPHIV cohorts, we used heterosexually active individuals at increased risk for HIV as the reference group because they had the highest projected life expectancy among CDC-defined HIV acquisition risk groups. Female youth with PHIV who met the definition of having average risk for HIV infection were projected to have a 6.7-year life expectancy gain compared with female youth with PHIV who met the definition of heterosexually active individuals at increased risk for HIV infection; those who met the definition of people WID were projected to have a 8.9-year life expectancy loss (Figure 3C). Among female youth with NPHIV, people WID had a projected life expectancy loss of 8.7 years (Figure 3D) compared with heterosexually active individuals at increased risk for HIV infection; a marked improvement after age 45 years reflects the relative mortality ratio age threshold.

### Ideal HIV Care Scenario

**Figure 4.**
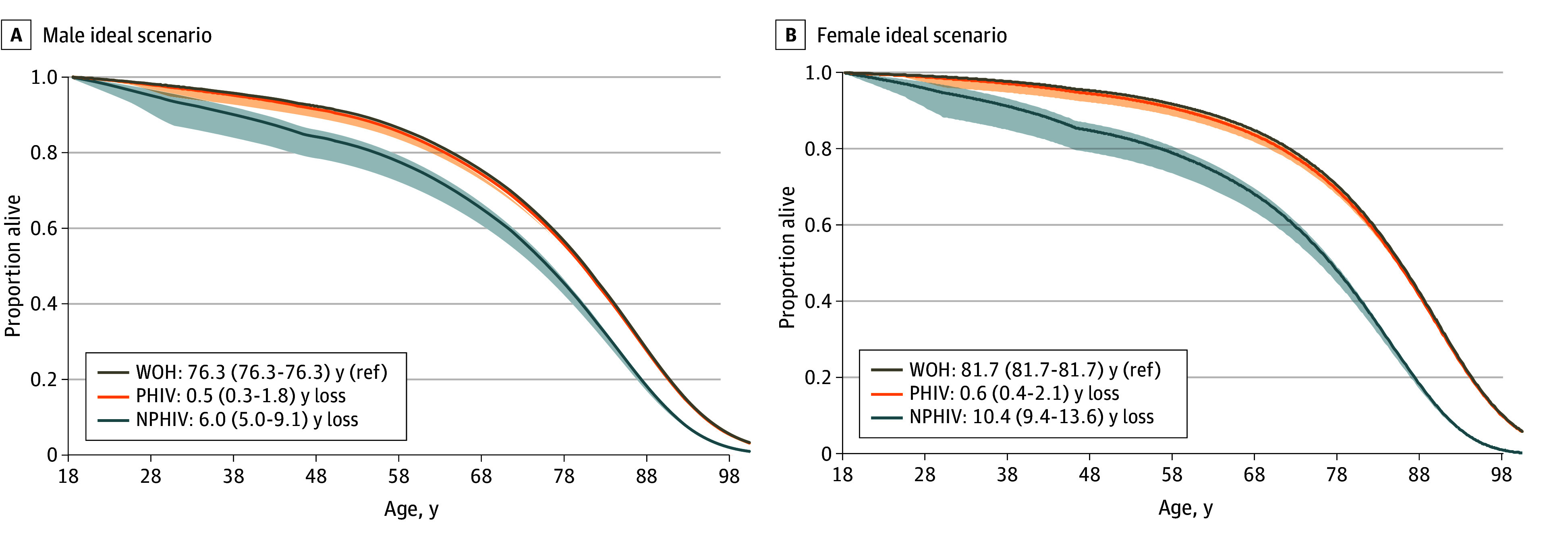
Projected Survival Over Time at Age 18 Years by Modeled Cohort Within an Ideal Care Scenario The first value reported underneath te curves is total mean life expectancy for the reference group (youth without HIV [WoH]); the other numbers show life expectancy losses compared with that reference group. The shaded areas represent the range of model results when varying adolescent HIV-related mortality input data through their 95% CIs. In the legend, the lower and upper estimates of life expectancy and life expectancy loss are shown in parentheses. NPHIV indicates HIV acquired nonperinatally; PHIV, HIV acquired perinatally; ref, reference.

In the ideal HIV care scenario, projected life expectancy losses for youth with PHIV compared with youth without HIV improved (Figure 4; 0.5 years for male individuals and 0.6 years for female individuals in the ideal care scenario vs 10.4 years for male individuals and 11.8 years for female individuals in the current care scenario). While life expectancy losses also improved for youth with NPHIV compared with youth without HIV with receipt of ideal HIV care, differences in life expectancy persisted (6.0 years for male individuals, 10.4 years for female individuals). To understand whether these projected persistent differences in life expectancy among youth with NPHIV compared with youth without HIV were due to non–HIV-related factors, we modeled a youth without HIV cohort with the same HIV acquisition risk group distribution as youth with NPHIV. Projected life expectancy losses among youth with NPHIV receiving the ideal care scenario decreased substantially compared with risk group–matched youth without HIV (1.3 years for male and female youth).

In eFigures 1 and 2 in Supplement 1, we report the effect of varying HIV-related and non–HIV-related mortality inputs through their 95% CIs. When we did not use income level to distinguish between heterosexually active individuals at increased risk for HIV infection and those at average risk, projected life expectancy was higher, with the most substantial improvement occurring among female youth with NPHIV (68.0 vs 62.1 years in the primary analyses; eFigure 3 in Supplement 1). eFigure 4 in Supplement 1 reports sex at birth–stratified life expectancy for different age thresholds of relative mortality ratios.

## Discussion

Using an adolescent-focused HIV microsimulation model, we projected lower life expectancy for youth with HIV compared with youth without HIV. While there is uncertainty around our life expectancy estimates due to considerable variability in the adolescent HIV-related mortality inputs, we projected youth with PHIV to have higher life expectancy than youth with NPHIV. We found that life expectancy losses for youth with PHIV were primarily informed by HIV disease, treatment, and care parameters. For youth with NPHIV, their life expectancy gap was associated with HIV-related parameters and the different distributions of sex at birth and HIV acquisition risk between cohorts. Youth with NPHIV had the highest proportion of male individuals, contributing to their lower overall projected life expectancy compared with the more sex-balanced youth with PHIV and youth without HIV cohorts. Lower life expectancy projections for male vs female individuals are consistent with overall US population estimates.^42^ Furthermore, the youth with NPHIV cohort only included individuals in CDC-defined HIV acquisition risk groups, all of which have higher mortality rates in the US than people at average risk for HIV acquisition. If we had assumed higher proportions of individuals in CDC-defined HIV acquisition risk groups for youth with PHIV than in the primary analysis, the gaps between youth with NPHIV and youth with PHIV would be diminished.

Compared with the 8-year to 13-year life expectancy gap estimated between adults with and without HIV,^8,9,10^ we projected life expectancy gaps of 11.0 years and 17.5 years for youth with PHIV and youth with NPHIV, respectively, compared with youth without HIV. We modeled cohorts of youth with HIV based on the best available data on current HIV treatment and care, with imperfect ART adherence and retention in care, in contrast to some studies that have used a life table approach^9^ that may consider mortality rates only among people not lost to follow-up. Our model specifically simulates deaths that may occur during time spent not receiving care and ART, which is the reality for many US youth with HIV.^43,44^ Our results align with data showing poorer HIV outcomes for youth with HIV compared with adults with HIV in the US^43^ and highlight the unique care needs of youth as they age into adulthood with HIV.

Given our assumptions of HIV acquisition risk group matching, while receiving ideal care, youth with PHIV achieved a similar life expectancy as youth without HIV (loss of 0.5 years for male individuals and 0.6 years for female individuals), highlighting the significance of addressing challenges to life-long ART adherence and care retention. In contrast, even while receiving ideal HIV care, a large gap in life expectancy persisted for youth with NPHIV (loss of 8.5 years). This persistent gap decreased when comparing youth with NPHIV with an HIV acquisition risk group–matched cohort of youth with WoH (loss of 1.3 years for male and female individuals), indicating that 7.2 years of life expectancy loss was due to non–HIV-related factors. Interventions are needed that address systemic and social factors (eg, poverty, housing, insurance eligibility, transportation, and a fragmented health care system) that contribute to disparities in non–HIV-related mortality, as well as those that contribute to barriers specifically in HIV care (eg, stigma). Although a full consideration of structural racism is beyond the scope of this analysis, closing the gap in life expectancy between youth with and without HIV requires dismantling racist practices and policies.^45,46,47^ The remaining 1.3 years of life expectancy may be due to HIV-related factors outside treatment optimization. Due to variability and/or incomplete data, we did not explicitly account for age-related and HIV-related noncommunicable diseases, multimorbidity, premature aging, toxic effects of ART, and time-varying retention after the transition to receiving adult care. These factors likely contribute to life expectancy among youth with HIV and may have led us to overestimate life expectancy, particularly for youth with PHIV who have longer exposure to HIV and ART.

Our experience developing an adolescent-specific microsimulation model highlighted the need for longitudinal data for youth with HIV, including reporting of cause of death rather than all-cause mortality,^9,33,48^ to better understand the contributions of comorbidities and disease progression to life expectancy. It also highlighted the importance of disaggregated outcome data for youth with PHIV and youth with NPHIV. We acknowledge the challenges in capturing mode of HIV acquisition, such as disclosure of parental HIV infection with documentation of perinatal infection, delays in diagnoses of HIV among youth, and the lack of routine capture of these data during the transition from pediatric to adult care. Any opportunities that allow for collection of these data would help efforts to identify focused interventions for youth, especially for the first generation of youth with PHIV who may not benefit equally from interventions that are targeted toward youth with NPHIV.

### Limitations

This analysis had several limitations. First, HIV mortality rates among adolescents not receiving care were derived from participants who spent time not receiving ART while remaining engaged in ATN/IMPAACT studies and could thus underestimate true out-of-care risks. This would lead our life expectancy projections to be optimistic for youth with PHIV and youth with NPHIV. However, while not receiving care, HIV-related mortality rates among adults not receiving ART were based on pre–ART-era data. Substantial changes have subsequently occurred in the population (eg, less HCV coinfection) and available care (eg, for critical illness). While we calibrated these inputs to more recent data,^31^ the adult HIV-related mortality inputs would lead our life expectancy projections to be more conservative for youth with PHIV and youth with NPHIV. Second, data for HIV-related clinical events and HIV-related mortality were not sex stratified, although published reports do not show a clear contribution of sex at birth.^8,49^ Third, while we tried to capture differences in treatment histories between youth with PHIV and youth with NPHIV, there are many factors that contribute to HIV treatment response. Fourth, in the absence of data to inform non–HIV-related mortality among those at increased risk for HIV, we used income as a proxy and varied this assumption in sensitivity analyses.^16^ Finally, we did not account for COVID-19–related mortality,^42^ which would lead us to overestimate life expectancy. Acknowledging these limitations, particularly variations in projections that may arise from data input uncertainty, this analysis nonetheless may provide valuable insight into critical factors that contribute to life expectancy for youth with HIV.

## Conclusions

This adolescent-focused microsimulation model of HIV disease and treatment was developed to project life expectancy among youth with HIV in the US. Within the current HIV treatment and care context, youth with PHIV and youth with NPHIV had lower model-projected life expectancy compared with youth without HIV. Projected differences were larger for youth with NPHIV compared with YPHIV; however, projected life expectancy estimates were variable due to uncertainty in HIV-related mortality. For youth with PHIV, there may be opportunities to close the life expectancy gap with youth without HIV by improving HIV treatment adherence and retention in care. To improve life expectancy for youth with NPHIV, in addition to optimizing HIV care, it will be necessary to identify interventions that address the structural and social factors that contribute to disparities in mortality by sex at birth as well as behaviors that increase risk for HIV infection.

## Notes

Supplement 1. eMethods. eTable 1. Race/ethnicity distributions for YHIV from different sources eTable 2. Additional base-case input parameters for a simulation model to estimate life expectancy among youth with HIV in the United States eTable 3. Model inputs: age and CD4-stratified monthly HIV-related mortality probabilities derived from ATN and IMPAACT data eTable 4. Age and CD4-stratified monthly HIV-related mortality rates per 100PY observed in ATN and IMPAACT data (before regression models and calibration) eTable 5. Microsimulation-model-projected person-time by age, CD4, and current ART use for adolescents eTable 6. Microsimulation-model-projected and expected HIV-related mortality rates by age for adolescents eTable 7. Microsimulation-model-projected person-time by age, CD4, and current ART use for adults eTable 8. Microsimulation-model-projected and expected HIV-related mortality rate by age for adults eTable 9. Microsimulation-model-projected and CDC-reported HIV-related mortality rate for US adults with HIV eTable 10. Sex-stratified monthly non-HIV-related mortality probabilities based on US mortality and population data, 2015 eTable 11. Relative mortality ratios, stratified by age and race/ethnicity eTable 12. Relative mortality ratios, stratified by sex, age, and risk for HIV acquisition eTable 13. Base-case life expectancy and life expectancy loss, stratified by sex assigned at birth, modeled cohort, and risk for HIV acquisition eTable 14. Monthly probabilities of race/ethnicity-adjusted non-HIV-related mortality of modeled cohorts, by age and sex eTable 15. Monthly probabilities of race/ethnicity- and risk-adjusted non-HIV-related mortality of modeled cohorts by age and sex eTable 16. Monthly probabilities of non-HIV-related mortality of modeled cohorts of men who have sex with men, by age and sex eTable 17. Monthly probabilities of non-HIV-related mortality of modeled cohorts of heterosexually active individuals at increased risk for HIV infection, by age and sex eTable 18. Monthly probabilities of non-HIV-related mortality of modeled cohorts of people who ever injected drugs, by age and sex eFigure 1. Projected survival from age 18 years, by sex, and 95% confidence intervals for adult HIV-related mortality inputs eFigure 2. Projected survival from age 18 years, by sex, and risk for HIV acquisition and 95% confidence intervals for relative mortality ratios for HIV acquisition risk eFigure 3. Projected survival from age 18 years, by sex, and sensitivity analysis using non-HIV-related mortality for individuals with average risk for HIV infection in place of non-HIV-related mortality from individuals with income below the federal poverty level eFigure 4. Projected survival from age 18 years, by sex, and age cutoffs for relative mortality ratios for HIV acquisition risk eReferences

Supplement 2. Data sharing statement
