# Supplementary material for: Projected Life Expectancy for Adolescents With HIV in the US
Source: JAMA Health Forum. 2024 May 10;5(5):e240816. doi: 10.1001/jamahealthforum.2024.0816 (PMC11087843; doi:10.1001/jamahealthforum.2024.0816)
Supplement: Supplement 1. — eMethods. eTable 1. Race/ethnicity distributions for YHIV from different sources eTable 2. Additional base-case input parameters for a simulation model to estimate life expectancy among youth with HIV in the United States eTable 3. Model inputs: age and CD4-stratified monthly HIV-related mortality probabilities derived from ATN and IMPAACT data eTable 4. Age and CD4-stratified monthly HIV-related mortality rates per 100PY observed in ATN and IMPAACT data (before regression models and calibration) eTable 5. Microsimulation-model-projected person-time by age, CD4, and current ART use for adolescents eTable 6. Microsimulation-model-projected and expected HIV-related mortality rates by age for adolescents eTable 7. Microsimulation-model-projected person-time by age, CD4, and current ART use for adults eTable 8. Microsimulation-model-projected and expected HIV-related mortality rate by age for adults eTable 9. Microsimulation-model-projected and CDC-reported HIV-related mortality rate for US adults with HIV eTable 10. Sex-stratified monthly non-HIV-related mortality probabilities based on US mortality and population data, 2015 eTable 11. Relative mortality ratios, stratified by age and race/ethnicity eTable 12. Relative mortality ratios, stratified by sex, age, and risk for HIV acquisition eTable 13. Base-case life expectancy and life expectancy loss, stratified by sex assigned at birth, modeled cohort, and risk for HIV acquisition eTable 14. Monthly probabilities of race/ethnicity-adjusted non-HIV-related mortality of modeled cohorts, by age and sex eTable 15. Monthly probabilities of race/ethnicity- and risk-adjusted non-HIV-related mortality of modeled cohorts by age and sex eTable 16. Monthly probabilities of non-HIV-related mortality of modeled cohorts of men who have sex with men, by age and sex eTable 17. Monthly probabilities of non-HIV-related mortality of modeled cohorts of heterosexually active individuals at increased risk for HIV infection, by age and sex eT [file jamahealthforum-e240816-s001.pdf]

Supplemental Online Content

Neilan AM, Ufio OL, Brenner IR, et al. Projected life expectancy for adolescents with HIV in the US. *JAMA Health Forum*. 2024;5(4):e240816. doi:10.1001/jamahealthforum.2024.0816

eMethods.

eTable 1. Race/ethnicity distributions for YHIV from different sources

eTable 2. Additional base-case input parameters for a simulation model to estimate life expectancy among youth with HIV in the United States

eTable 3. Model inputs: age and CD4-stratified monthly HIV-related mortality probabilities derived from ATN and IMPAACT data

eTable 4. Age and CD4-stratified monthly HIV-related mortality rates per 100PY observed in ATN and IMPAACT data (before regression models and calibration)

eTable 5. Microsimulation-model-projected person-time by age, CD4, and current ART use for adolescents

eTable 6. Microsimulation-model-projected and expected HIV-related mortality rates by age for adolescents

eTable 7. Microsimulation-model-projected person-time by age, CD4, and current ART use for adults

eTable 8. Microsimulation-model-projected and expected HIV-related mortality rate by age for adults

eTable 9. Microsimulation-model-projected and CDC-reported HIV-related mortality rate for US adults with HIV

eTable 10. Sex-stratified monthly non-HIV-related mortality probabilities based on US mortality and population data, 2015

eTable 11. Relative mortality ratios, stratified by age and race/ethnicity

eTable 12. Relative mortality ratios, stratified by sex, age, and risk for HIV acquisition

eTable 13. Base-case life expectancy and life expectancy loss, stratified by sex assigned at birth, modeled cohort, and risk for HIV acquisition

eTable 14. Monthly probabilities of race/ethnicity-adjusted non-HIV-related mortality of modeled cohorts, by age and sex

eTable 15. Monthly probabilities of race/ethnicity- and risk-adjusted non-HIV-related mortality of modeled cohorts by age and sex

© 2024 Neilan AM et al. *JAMA Health Forum.*

eTable 16. Monthly probabilities of non-HIV-related mortality of modeled cohorts of men who have sex with men, by age and sex

eTable 17. Monthly probabilities of non-HIV-related mortality of modeled cohorts of heterosexually active individuals at increased risk for HIV infection, by age and sex

eTable 18. Monthly probabilities of non-HIV-related mortality of modeled cohorts of people who ever injected drugs, by age and sex

eFigure 1. Projected survival from age 18 years, by sex, and 95% confidence intervals for adult HIV-related mortality inputs

eFigure 2. Projected survival from age 18 years, by sex, and risk for HIV acquisition and 95% confidence intervals for relative mortality ratios for HIV acquisition risk

eFigure 3. Projected survival from age 18 years, by sex, and sensitivity analysis using non-HIV-related mortality for individuals with average risk for HIV infection in place of non-HIV-related mortality from individuals with income below the federal poverty level

eFigure 4. Projected survival from age 18 years, by sex, and age cutoffs for relative mortality ratios for HIV acquisition risk

eReferences

This supplemental material has been provided by the authors to give readers additional information about their work.

eMETHODS

Distribution of race/ethnicity among modeled cohorts

The race/ethnicity distribution of the cohorts was informed by United States (US) Centers for Disease Control and Prevention (CDC) data among youth with HIV (YHIV) ages 13 to 24 years (Manuscript Table 1). We used the same distribution of race/ethnicity for both youth with HIV acquired perinatally (YPHIV) and youth with HIV acquired non-perinatally (YNPHIV), which have similar reported race/ethnicity in national cohort datasets (ATN, IMPAACT, HIV Research Network), as well as the CDC data (eTable 1) [2, 14-15].

Mortality input derivation and validation overview

HIV-related and non-HIV-related mortality were distinguished in model inputs. Risk for HIV-related mortality depended on age, CD4 count and antiretroviral status while risk for non-HIV-related mortality depended on age, sex, race/ethnicity, and risk for HIV acquisition.

Adolescent Medicine Trial Network for HIV/AIDS Intervention (ATN) and International Maternal Pediatric Adolescent AIDS Clinical Trials (IMPAACT) Network[15, 50] data were used to derive monthly probabilities of HIV-related mortality separately for YPHIV and YNPHIV from ages 18 to <30 years ("adolescent" inputs, eTable 3). Multicenter AIDS Cohort Study (MACS)[21] and HIV-CAUSAL Collaboration [22] data informed off-ART HIV-related mortality probabilities from age 30 years onward; published North American AIDS Cohort Collaboration on Research and Design (NA-ACCORD) data[23-24] informed on-ART HIV-related mortality probabilities from age 30 years onward ("adult" inputs, Manuscript Table 1). Overall adult HIV-related mortality was calibrated to data from the CDC[37].

We calculated monthly non-HIV-related mortality probabilities using a life table approach. We calculated sex-stratified HIV-deleted life tables as described elsewhere[51], using cause-specific

© 2024 Neilan AM et al. *JAMA Health Forum.*

mortality data[29], and population size estimates by age and sex[30, 52] (eTable 10). To generate monthly race/ethnicity-adjusted non-HIV-related mortality probabilities, we calculated race/ethnicity-specific HIV-deleted life tables and weighted by the race/ethnicity distribution among the YHIV cohorts. These race/ethnicity-adjusted, sex-stratified mortality rates were then adjusted by risk for HIV acquisition by applying risk factor-specific relative mortality ratios (RMRs) weighted by the distribution of risk for HIV acquisition among YPHIV and YNPHIV, respectively. Categories for risk for HIV acquisition included men who have sex with men (MSM), heterosexually active individuals at increased risk for HIV infection, and people who ever injected drugs (PWID). Because we found limited data to inform non-HIV-related mortality among heterosexually active individuals at increased risk for HIV infection, we used non-HIV-related mortality among individuals with incomes below the federal poverty level as a proxy[16, 53].

HIV-related mortality

Adolescent input derivation

To derive adolescent HIV-related mortality model inputs, we used age-, CD4- and viral-load-/antiretroviral-therapy-stratified ATN and IMPAACT data[15, 50]. In the ATN and IMPAACT datasets, we defined HIV-related mortality as that attributed to CDC or World Health Organization (WHO)-defined HIV-related events. We also included suicide in this category. The observed mortality rates in the original ATN/IMPAACT data can be found in eTable 4.

Given that mortality events were not observed within some age, CD4 count, and viral load/antiretroviral therapy (ART) strata in the ATN and IMPAACT data due to limited person-time of follow-up in the data, we used a generalized linear regression model with a log gamma distribution in SAS Software[53] to obtain predicted mortality rates by age, CD4, and viral load/ART strata which were then converted to monthly probabilities of adolescent HIV-related mortality for the microsimulation model.

© 2024 Neilan AM et al. *JAMA Health Forum.*

These regression-estimated inputs were then calibrated to the overall mortality rate observed in the ATN and IMPAACT data by using the ratio of the overall mortality rate calculated from the ATN and IMPAACT data/the overall mortality rate observed from the regression model. This ratio was used to consistently scale down the regression-estimated age-, CD4-, and viral-load-/ART-stratified inputs to produce the final derived inputs for adolescent HIV-related mortality (eTable 3).

Adolescent input validation

We then assessed the difference between microsimulation-model-projected HIV-related mortality rates and the source data from which it was derived[54]. We ran the simulation and extracted person-time for each relevant age group (18-24 and 25-30 years) stratified by CD4 and current ART use (eTable 5).

Then, we multiplied these percentages in eTable 5 by the corresponding mortality probabilities (eTable 3) to calculate an "expected" HIV-related mortality rate. Finally, we compared that expected rate to the microsimulation-model-projected HIV-related mortality rate from the same stratum using the ratio between the two values (such that a ratio equal to 1 would represent perfect agreement between the mortality rates). We also performed the same calculations using the original, observed mortality rates from the ATN/IMPAACT data (eTable 4), to assess the differences between the final model inputs and the observed mortality rates (eTable 6).

The modeled HIV-related mortality rates varied from the expected HIV-related mortality rates by a margin of ~1% or less and fell within the 95% confidence interval (CI) bounds of the observed HIV-related mortality rates from the ATN and IMPAACT data (rightmost column of eTable 6).

© 2024 Neilan AM et al. *JAMA Health Forum.*

Adult input derivation

To calculate adult HIV-related mortality, we used MACS[21] and HIV-CAUSAL[22] data for off-ART mortality rates stratified by CD4 count. We used the mortality ratio comparing each CD4 strata to the CD4 <50 strata from the MACS dataset and scaled the off-ART HIV-related mortality rate to the HIV-CAUSAL off-ART mortality rate to reflect the change in mortality in the current era. We used data from published NA-ACCORD studies to calculate on-ART mortality rates by CD4 cell count, which is based on patients who entered HIV care between 2011 and 2017[23-24].

Adult input validation

We again ran the microsimulation model and extracted person-time for the 'adult' portion of the model (age >30 years) stratified by CD4 and current ART use (eTable 7).

Because the adult HIV-related mortality consists of both opportunistic-infection (OI)-related mortality and 'chronic' HIV mortality (i.e., HIV-related mortality of non-OI causes), we adjusted these percentages to account for the expected amount of time that an individual had an opportunistic infection within each CD4 strata. Then we multiplied these percentages by the corresponding mortality probabilities (Manuscript Table 1) to calculate an "expected" HIV-related mortality rate. Finally, we compared that expected rate to the modeled HIV-related mortality rate for ages >30 years using the ratio between the two values (such that a ratio equal to 1 would represent perfect agreement between the mortality rates) (eTable 8). The microsimulation modeled HIV-related mortality rates varied from the expected HIV related mortality rates by a margin of ~1% or less.

After validating our inputs, we compared our microsimulation-model-projected HIV-related mortality rate to an external source to assess the magnitude of the difference and assess face validity. To do this, we extracted the modeled chronic HIV-related mortality (as in eTable 8), this

© 2024 Neilan AM et al. *JAMA Health Forum.*

time combining it with the modeled OI-related mortality to find the overall HIV-related mortality. We then compared this to the HIV-related mortality reported by the CDC[37], and calculated the absolute and percent difference in the values (eTable 9). The modeled HIV-related mortality rate was lower than the CDC-reported rate. The absolute difference in rates would represent a total difference of around 1,000 HIV-related deaths annually in the US out of approximately 16,000 annual all-cause deaths among 1.2 million people with HIV in the US[37].

Relative mortality ratios

We used cause-specific mortality data[29], and population size estimates by age, sex, and race/ethnicity[30, 52] to estimate race/ethnicity-specific mortality rates and average population mortality rates. Subsequently, we calculated the RMRs stratified by race/ethnicity by comparing the mortality rate observed in each race to the average population mortality rate (eTable 11). We used publicly available data on mortality from the 2001 to 2014 National Health and Nutrition Examination Surveys (NHANES)[44] and their Linked Mortality Files (LMF)[55] to generate RMRs for the HIV acquisition risk categories. LMFs link survey data from NHANES with death certificate records from the National Death Index. We then derived RMRs stratified by risk for HIV acquisition by comparing observed mortality of various risk for HIV acquisition cohorts to the mortality of those without the risk category (eTable 12). The corresponding RMRs were then applied to the baseline monthly non-HIV-related mortality rates from US Life Tables to adjust for race/ethnicity and risk for HIV acquisition in our modeled cohorts. Once RMRs were calculated that factored in age, sex, race/ethnicity, and risk factor group, they were combined with the baseline non-HIV-related mortality using the relevant distribution of individuals to reach the final mortality probability at any age for any given person in the model.

© 2024 Neilan AM et al. *JAMA Health Forum.*

Age-adjusted relative mortality ratios

To reflect age-related changes in non-HIV-related mortality risks related to being assigned to one of the CDC-defined risk for HIV acquisition categories (MSM, heterosexually active individuals at increased risk for HIV infection, or PWID), we derived RMRs separately for individuals below and over 45 years of age for each of these categories, for both the risk for HIV acquisition and race-stratified RMRs. This age threshold generally resulted in lower RMRs and consequently lower mortality rates on average for individuals older than 45 years (Tables S11 and S12).

In some cases, the age threshold resulted in significantly lower mortality rates for individuals older than 45 years. For example, PWID had significantly lower mortality rates after age 45 years compared to before age 45 years. This is a result of the lower incidence of drug use at older ages combined with the survivor bias among by those who lived to that point. Due to the high rates of premature mortality that PWID experience, there is a difference in survival before and after age 45 years. This change in survival after age 45 years is visible from the inflection points in the projected survival curves for PWID (Manuscript Figure 3). We conducted sensitivity analyses, using age 55 years as the threshold and eliminating an age threshold altogether. The inflection in the survival curve remained when we used age 55 years as the threshold and was eliminated when we eliminated the age threshold (eFigure 4). However, we elected to use age 45 years to ensure that there was adequate sample size in both age strata. The overall results remained consistent across these sensitivity analyses, with the PWID cohort having the lowest projected survival compared to other risk cohorts.

Socioeconomic status as a proxy for HIV acquisition risk among heterosexually active individuals

Because there is no single method to ascertain which heterosexually active individuals are at increased risk for HIV infection, the initial cycle of the National HIV Behavioral Surveillance system[56] conducted a pilot study using venue-based sampling and respondent-driven sampling to determine an optimal sampling strategy[57]. This initial study, based on 2006-2007 data, found an association between socioeconomic status and HIV prevalence, postulating that adverse social conditions associated with economic status could increase the risk of HIV infection including sexual exploitation, unstable sexual partnerships, limited access to health care and preventive services and socioeconomic segregation confining individuals to sexual networks with high underlying rates of HIV and other sexually transmitted infections.

Since that time, in each of its reporting cycles (2007, 2010, 2013, 2016 and 2019), the National HIV Behavioral Surveillance report has collected data among heterosexually active adults at increased risk for HIV infection, using socioeconomic measures as a marker of increased risk for HIV. The most recent 2019 report inclusion criteria included participants classified as low income (instead of participants classified as low socioeconomic status), defined as achieving no more than high school education or having income at or below the federal poverty level, calculated by the Health and Human Services (HHS) poverty guidelines[16]. This change was made to account for substantial differences in cost of living across cities.

We defined low socioeconomic status as those with poverty income ratio (PIR) less than one. The PIR is the midpoint of family income divided by the calendar-year-specific poverty threshold defined by the US Census Bureau[25].

© 2024 Neilan AM et al. *JAMA Health Forum.*

Non-HIV-related mortality

The distributions of race/ethnicity and risk for HIV acquisition among YHIV were used to calculate non-HIV-related mortality by the following process. We first calculated overall and race/ethnicity-specific HIV-deleted US life tables as described elsewhere[51], using cause-specific mortality data and population size estimated by age, sex, and race/ethnicity (eTable 10). We then calculated race/ethnicity-based RMRs from this data (eTable 11). Then, using the race/ethnicity distribution for the population of YHIV (Table 1) and the race/ethnicity-based RMRs, we calculated the race-adjusted non-HIV-related mortality (eTable 14). We conducted a similar process for the distribution of risk factors for HIV infection. Starting with the race-adjusted non-HIV-related mortality, we used risk RMRs calculated from NHANES data (eTable 14) and the risk distribution for YPHIV/YWoH and YNPHIV to calculate the race- and risk-adjusted non-HIV-related mortality (eTable 15). The overall race/ethnicity distribution for each cohort was 61% Black or African American, 24% Hispanic or Latino, and 15% White; however, each of the three cohorts were multiplied by different distributions of risk for HIV acquisition. The distributions of HIV acquisition risk factors for modeled YPHIV and YWoH cohorts were set to match the general population, with both modeled with 6% / 0% MSM (male/female), 14% / 9% heterosexually active individual at increased risk for HIV infection, 77% / 89% average risk for HIV infection, 3% / 2% PWID. Meanwhile, the YNPHIV cohort had a greater proportion of MSM and PWID, with males/females modeled as MSM (93% / 0%), heterosexually active individual at increased risk for HIV infection (3% / 90%), average risk for HIV infection (0% / 0%), and PWID (4% / 10%).

© 2024 Neilan AM et al. *JAMA Health Forum.*

eTable 1. Race/ethnicity distributions for YHIV from different sources

Cohort	Black, %	Hispanic, %	White, %	Other race/ethnicity, %	Source
YPHIV	62	28	10	-	ATN/IMPAACT[15]
YPHIV	63	20	16	1	HIV Research Network[14]
YPHIV	62	19	11	8	CDC[2]
YNPHIV	58	32	10	-	ATN/IMPAACT[15]
YNPHIV	63	17	16	4	HIV Research Network[14]
YNPHIV	56	24	15	6	CDC[2]
All YHIV	57	23	14	7	CDC[2]
All YHIV (this analysis)	61	24	15	-	CDC[2]

ATN: Adolescent Medicine Trials Network for HIV/AIDS Interventions; **CDC:** Centers for Disease Control and Prevention; **IMPAACT:** International Maternal Pediatric Adolescent AIDS Clinical Trial Network; **YHIV:** youth with HIV; **YNPHIV:** youth with HIV acquired non-perinatally; **YPHIV:** youth with HIV acquired perinatally.

© 2024 Neilan AM et al. *JAMA Health Forum.*

eTable 2. Additional base-case input parameters for a simulation model to estimate life expectancy among youth with HIV in the United States

Base-case parameter	YPHIV	YNPHIV	Source
**HIV RNA setpoint distribution, % of cohort**			36
>100,000 copies/mL	25.1	25.1	
30,001-100,000	42.0	42.0	
10,001-30,000	20.9	20.9	
3,001-10,000	5.6	5.6	
501-3,000	6.4	6.4	
0-500	0	0	
**Adherence to ART, % of initial cohort**			13, 58
Adherence >90%	40	40	
Adherence 81-90%	27	27	
Adherence 71-80%	15	15	
Adherence 61-70%	8	8	
Adherence ≤60%	10	10	

ART: antiretroviral therapy; **RNA**: ribonucleic acid; **YNPHIV:** youth with HIV acquired non-perinatally; **YPHIV:** youth with HIV acquired perinatally.

© 2024 Neilan AM et al. *JAMA Health Forum.*

eTable 3. Model inputs: Age and CD4-stratified monthly HIV-related mortality probabilities derived from ATN and IMPAACT data

CD4 cell count stratum	YPHIV		YNPHIV		Source
	18 to <25y	25 to <30y	18 to <25y	25 to <30y	
		Off antiretroviral therapy			
>500 cells/μL	0.00003	0.00006	0.00054	0.00067	
201-500	0.00043	0.00094	0.00152	0.00262	
<200	0.00335	0.00575	0.00227	0.00719	
		On antiretroviral therapy			15
>500 cells/μL	0.00002	0.00004	0.00013	0.00016	
201-500	0.00031	0.00069	0.00040	0.00054	
<200	0.00256	0.00457	0.00059	0.00111	

ATN: Adolescent Medicine Trials Network for HIV/AIDS Interventions; **IMPAACT:** International Maternal Pediatric Adolescent AIDS Clinical Trials Network; **YNPHIV:** youth with HIV acquired non-perinatally; **YPHIV:** youth with HIV acquired perinatally **y:** years.

© 2024 Neilan AM et al. *JAMA Health Forum.*

eTable 4. Age and CD4-stratified monthly HIV-related mortality rates per 100PY observed in ATN and IMPAACT data (before regression models and calibration)

CD4 cell count stratum	YPHIV		YNPHIV		Source
	18 to <25y (95% CI)	25 to <35y (95% CI)	18 to <25y (95% CI)	25 to <35y (95% CI)	
		Off antiretroviral therapy			
>500 cells/µL	0.0 (0.0-8.44)	0.0 (0.0-111.82)	2.28 (0.07-12.72)	0.0 (0.0-55.07)	
201-500	0.0 (0.0-3.74)	0.0 (0.0-49.20)	0.0 (0.0-9.46)	0.0 (0.0-20.61)	
<200	7.55 (2.45-17.63)	7.63 (0.23-42.52)	0.0 (0.0-141.92)	32.26 (0.97-179.68)	15
		On antiretroviral therapy			
>500 cells/µL	0.0 (0.0-0.34)	0.0 (0.0-3.30)	0.35 (0.01-1.94)	0.0 (0.0-2.58)	
201-500	0.45 (0.09-1.32)	1.36 (0.04-7.56)	0.0 (0.0-4.08)	0.0 (0.0-5.49)	
<200	2.36 (1.02-4.65)	7.60 (2.07-19.47)	0.0 (0.0-29.29)	3.73 (0.11-20.78)	

ATN: Adolescent Medicine Trials Network for HIV/AIDS Treatment; **CI:** confidence interval; **IMPAACT:** International Maternal Pediatric Adolescent AIDS Clinical Trial network; **PY:** person-years; **YNPHIV:** youth with HIV acquired non-perinatally; **YPHIV:** youth with HIV acquired perinatally **y:** years.

© 2024 Neilan AM et al. *JAMA Health Forum.*

eTable 5. Microsimulation-model-projected person-time by age, CD4, and current ART use for adolescents

CD4 cell count stratum	Age 18-24 years		Age 25-30 years	
	Off ART, person-time, %	On ART, person-time, %	Off ART, person-time, %	On ART, person-time, %
**YPHIV**				
>500 cells/μL	13.5	47.4	12.4	44.5
200-500	11.6	15.0	13.1	7.7
<200	8.6	4.1	18.5	3.8
**YNPHIV**				
>500 cells/μL	9.6	48.4	9.5	44.4
200-500	16.3	16.6	13.1	10.8
<200	6.9	2.3	16.5	5.7

ART: antiretroviral therapy; **YNPHIV:** youth with HIV acquired non-perinatally; **YPHIV:** youth with HIV acquired perinatally.

© 2024 Neilan AM et al. *JAMA Health Forum.*

eTable 6. Microsimulation-model-projected and expected HIV-related mortality rates by age for adolescents

Age	Microsimulation-model-projected HIV-related mortality rate/100PY	Expected HIV-related mortality rate/100PY*	Ratio (microsimulation-model-projected/expected)	Observed HIV-related mortality/100PY (95% CI) in ATN/IMPAACT data
YPHIV				
18-24 years	0.5971	0.5993	0.9963	0.7 (0.4, 1.1)
25-30 years	1.7177	1.7315	0.9920	2.3 (0.8, 5.0) †
YNPHIV				
18-24 years	0.7264	0.7193	1.0099	0.4 (0.1, 1.5)
25-30 years	2.1666	2.1514	1.0071	0.8 (0.1, 2.7) †

*Based on final inputs using regression models derived from ATN/IMPAACT data. †These rates were for the 25-<35-year-old age stratum in the original dataset.
ATN: Adolescent Medicine Trials Network for HIV/AIDS Interventions; **IMPAACT:** International Maternal Pediatric Adolescent AIDS Clinical Trials Network; **PY:** person-years; **YNPHIV:** youth with HIV acquired non-perinatally; **YPHIV:** youth with HIV acquired perinatally.

© 2024 Neilan AM et al. *JAMA Health Forum.*

eTable 7. Microsimulation-model-projected person-time by age, CD4, and current ART use for adults

CD4 cell count stratum	>30 years	
	Off ART person-time, %	On ART person-time, %
**YPHIV**		
>500 cells/µL	9.8	61.5
350-500	2.9	4.0
200-350	3.9	3.5
100-200	3.5	2.0
50-100	2.1	0.9
<50	5.2	0.8
**YNPHIV**		
>500 cells/µL	9.6	61.4
350-500	2.9	4.2
200-350	4.0	3.6
100-200	3.6	2.0
50-100	2.2	0.9
<50	5.0	0.7

ART: antiretroviral therapy; **YNPHIV:** youth with HIV acquired non-perinatally; **YPHIV:** youth with HIV acquired perinatally.

© 2024 Neilan AM et al. *JAMA Health Forum.*

eTable 8. Microsimulation-model-projected and expected HIV-related mortality rate by age for adults

Age	Modeled HIV-related mortality rate/100PY	Expected HIV-related mortality rate/100PY	Ratio (modeled/expected)
YPHIV			
>30 years	0.2614	0.2615	0.9994
YNPHIV			
>30 years	0.2587	0.2564	1.0092

PY: person-years; **YNPHIV:** youth with HIV acquired non-perinatally; **YPHIV:** youth with HIV acquired perinatally.

eTable 9. Microsimulation-model-projected and CDC-reported HIV-related mortality rate for US adults with HIV

	Modeled HIV-related mortality rate/100PY for age >30 years	CDC reported HIV-related mortality rate/100PY (46)	Absolute difference	Percent difference
YPHIV	0.385	0.47	-0.085	-18.0%
YNPHIV	0.381	0.47	-0.089	-19.0%

CDC: Centers for Disease Control and Prevention; **PY:** person-years; **YNPHIV:** youth with HIV acquired non-perinatally; **YPHIV:** youth with HIV acquired perinatally.

© 2024 Neilan AM et al. *JAMA Health Forum.*

eTable 10. Sex-stratified monthly non-HIV-related mortality probabilities based on US mortality and population data, 2015

Age	Male	Female	Source
18 – 19 years	0.00007 - 0.00009	0.00003 - 0.00003	29, 30
20 – 24	0.00009 - 0.00012	0.00003 - 0.00004	
25 – 29	0.00012 - 0.00014	0.00005 - 0.00006	
30 – 39	0.00013 - 0.00018	0.00006 - 0.00010	
40 – 49	0.00018 - 0.00038	0.00011 - 0.00024	
50 – 59	0.00041 - 0.00089	0.00026 - 0.00053	
60 – 69	0.00094 - 0.00182	0.00055 - 0.00120	
70 – 79	0.00191 - 0.00430	0.00128 - 0.00313	
80 – 89	0.00477 - 0.01230	0.00347 - 0.00978	
90 – 99	0.01360 - 0.02622	0.01104 - 0.02549	
100	1.00	1.00	

This table contains non-HIV-related mortality for the US general population. This does not include the race/ethnicity distribution of US youth with HIV.

eTable 11. Relative mortality ratios, stratified by age and race/ethnicity

Race/ethnicity	Age ≤45y	Age >45y	Source
Black	1.590	1.013	Derived from age- sex-, and race-stratified mortality[29], and population size data[30,52]
Hispanic	0.722	0.585	
White	1.056	1.110	

Y: years

© 2024 Neilan AM et al. *JAMA Health Forum.*

eTable 12. Relative mortality ratios, stratified by sex, age, and risk for HIV acquisition

Risk for HIV acquisition	Age ≤45y (95% CI)	Age >45y (95% CI)	Source
Male			
Men who have sex with men	2.208 (1.014, 4.808)	1.407 (0.764, 2.590)	25
Heterosexually active individuals at increased risk for HIV infection	1.467 (0.873, 2.465)	1.975 (1.417, 2.752)	
Average risk for HIV infection	1.000 (1.000, 1.000)	1.000 (1.000, 1.000)	
People who ever injected drugs	3.542 (1.533, 8.180)	1.950 (1.006, 3.778)	
Female			
Heterosexually active individuals at increased risk for HIV infection	3.026 (1.640, 5.583)	2.037 (1.329, 3.122)	25
Average risk for HIV infection	1.000 (1.000, 1.000)	1.000 (1.000, 1.000)	
People who ever injected drugs	15.174 (5.317, 43.305)	1.613 (0.657, 3.959)	

CI: confidence interval; **y:** years.

© 2024 Neilan AM et al. *JAMA Health Forum.*

eTable 13. Base-case life expectancy and life expectancy loss, stratified by sex assigned at birth, modeled cohort, and risk for HIV acquisition

	Male		Female		Overall	
	Life expectancy	Loss	Life expectancy	Loss	Life expectancy	Loss
**By cohort**						
YWoH	76.3	Ref	81.7	Ref	79.0	Ref
YPHIV	65.9	10.4	69.9	11.8	68.0	11
YNPHIV	61.4	15	62.1	19.5	61.5	17.5
**By risk for HIV acquisition**						
YPHIV						
Average	67.2	Ref	70.7	Ref	69.1	Ref
MSM	62.5	4.7	-	-	62.5	6.6
HIH	61.6	5.6	64.0	6.7	62.9	6.2
PWID	58.2	9.1	55.2	15.6	56.6	12.5
YNPHIV						
MSM	61.6	Ref	-	-	61.6	Ref
HIH	60.7	0.9	63.0	Ref	61.0	0.5
PWID	57.3	4.3	54.4	8.7	56.9	4.7

HIH: heterosexually active individual at increased risk for HIV infection; **MSM:** men who have sex with men; **PWID:** people who ever injected drugs; **Ref:** reference group; **YNPHIV**: youth with HIV acquired non-perinatally; **YPHIV**: youth with HIV acquired perinatally; **YWoH**: youth without HIV.

© 2024 Neilan AM et al. *JAMA Health Forum.*

eTable 14. Monthly probabilities of race/ethnicity-adjusted non-HIV-related mortality of modeled cohorts, by age and sex

Age	YPHIV, YNPHIV, and YWoH	
	Male	Female
18 – 19 years	0.00009 - 0.00011	0.00004 - 0.00004
20 – 24	0.00012 - 0.00015	0.00004 - 0.00005
25 – 29	0.00015 - 0.00017	0.00006 - 0.00007
30 – 39	0.00016 - 0.00022	0.00008 - 0.00012
40 – 49	0.00022 - 0.00034	0.00013 - 0.00021
50 – 59	0.00036 - 0.00079	0.00023 - 0.00047
60 – 69	0.00083 - 0.00159	0.00049 - 0.00106
70 – 79	0.00167 - 0.00370	0.00113 - 0.00273
80 – 89	0.00409 - 0.00994	0.00302 - 0.00816
90 – 99	0.01087 - 0.01781	0.00913 - 0.01829
100	1.00	1.00

This table contains non-HIV-related mortality for youth with HIV adjusted for the race/ethnicity distribution specified in Table 1, using the relative mortality ratios in eTable 4. We also used this mortality as a proxy for the mortality of individuals with average risk for HIV.

YNPHIV: youth with HIV acquired non-perinatally; **YPHIV:** youth with HIV acquired perinatally; **YWoH:** youth without HIV.

eTable 15. Monthly probabilities of race/ethnicity- and risk-adjusted non-HIV-related mortality of modeled cohorts by age and sex

Age, years	YPHIV and YWoH		YNPHIV	
	Male	Female	Male	Female
18 – 19	0.00011 - 0.00013	0.00005 - 0.00006	0.00020 - 0.00024	0.00016 - 0.00018
20 – 24	0.00014 - 0.00018	0.00006 - 0.00007	0.00026 - 0.00033	0.00018 - 0.00022
25 – 29	0.00018 - 0.00020	0.00008 - 0.00010	0.00033 - 0.00038	0.00024 - 0.00030
30 – 39	0.00019 - 0.00026	0.00011 - 0.00017	0.00036 - 0.00049	0.00032 - 0.00050
40 – 49	0.00027 - 0.00040	0.00018 - 0.00027	0.00037 - 0.00069	0.00033 - 0.00080
50 – 59	0.00043 - 0.00093	0.00026 - 0.00052	0.00053 - 0.00114	0.00047 - 0.00094
60 – 69	0.00098 - 0.00185	0.00054 - 0.00115	0.00120 - 0.00230	0.00099 - 0.00212
70 – 79	0.00193 - 0.00417	0.00122 - 0.00293	0.00241 - 0.00531	0.00226 - 0.00546
80 – 89	0.00459 - 0.01063	0.00323 - 0.00849	0.00587 - 0.01415	0.00603 - 0.01619
90 – 99	0.01156 - 0.01814	0.00947 - 0.01848	0.01546 - 0.02510	0.01810 - 0.03558
100	1.00	1.00	1.00	1.00

This table contains base-case non-HIV-related mortality used for the runs to produce Figure 2 in the main text of the manuscript. Note that mortality for males tended to be higher than for females, and mortality for YNPHIV tended to be higher than for YPHIV/YWoH.

YNPHIV: youth with HIV acquired non-perinatally; **YPHIV:** youth with HIV acquired perinatally; **YWoH:** youth without HIV.

© 2024 Neilan AM et al. *JAMA Health Forum.*

eTable 16. Monthly probabilities of non-HIV-related mortality of modeled cohorts of men who have sex with men, by age and sex

Age, years	YPHIV, YNPHIV, and YWoH
	Male
18 – 19	0.00020 - 0.00024
20 – 24	0.00026 - 0.00032
25 – 29	0.00032 - 0.00037
30 – 39	0.00036 - 0.00048
40 – 49	0.00036 - 0.00069
50 – 59	0.00051 - 0.00111
60 – 69	0.00117 - 0.00224
70 – 79	0.00235 - 0.00520
80 – 89	0.00575 - 0.01395
90 – 99	0.01525 - 0.02496
100	1.00

This table contains base-case non-HIV-related mortality for modeled MSM cohorts. It was calculated using the race-adjusted non-HIV-mortality, and the RMR for MSM calculated from National Health and Nutrition Examination Survey data[25].

MSM: men who have sex with men; **YNPHIV:** youth with HIV acquired non-perinatally; **YPHIV:** youth with HIV acquired perinatally; **YWoH:** youth without HIV.

eTable 17. Monthly probabilities of non-HIV-related mortality of modeled cohorts of heterosexually active individuals at increased risk for HIV infection, by age and sex

Age	YPHIV, YNPHIV, and YWoH	
	Male	Female
18 – 19 years	0.00013 - 0.00016	0.00011 - 0.00013
20 – 24	0.00017 - 0.00021	0.00013 - 0.00016
25 – 29	0.00022 - 0.00025	0.00017 - 0.00021
30 – 39	0.00024 - 0.00032	0.00023 - 0.00037
40 – 49	0.00032 - 0.00066	0.00033 - 0.00062
50 – 59	0.00072 - 0.00156	0.00048 - 0.00096
60 – 69	0.00165 - 0.00315	0.00100 - 0.00215
70 – 79	0.00329 - 0.00729	0.00230 - 0.00555
80 – 89	0.00806 - 0.01953	0.00614 - 0.01655
90 – 99	0.02135 - 0.03486	0.01852 - 0.03690
100	1.00	1.00

This table contains base-case non-HIV-related mortality for modeled cohorts of heterosexually active individuals with increased risk for HIV infection. It was calculated using the race/ethnicity-adjusted non-HIV-mortality, and the relative mortality ratios for individuals with incomes below the calendar year-specific federal poverty level calculated from National Health and Nutrition Examination Survey data[25]. We used this mortality as a proxy for the mortality of heterosexually active individuals increased risk for HIV infection.

YNPHIV: youth with HIV acquired non-perinatally; **YPHIV:** youth with HIV acquired perinatally; **YWoH:** youth without HIV.

© 2024 Neilan AM et al. *JAMA Health Forum.*

eTable 18. Monthly probabilities of non-HIV-related mortality of modeled cohorts of people who ever injected drugs, by age and sex

Age	YPHIV, YNPHIV, and YWoH	
	Male	Female
18 – 19 years	0.00032 - 0.00038	0.00057 - 0.00064
20 – 24	0.00041 - 0.00052	0.00065 - 0.00080
25 – 29	0.00052 - 0.00060	0.00087 - 0.00107
30 – 39	0.00057 - 0.00078	0.00115 - 0.00186
40 – 49	0.00049 - 0.00110	0.00027 - 0.00310
50 – 59	0.00071 - 0.00154	0.00038 - 0.00076
60 – 69	0.00162 - 0.00311	0.00079 - 0.00170
70 – 79	0.00325 - 0.00720	0.00182 - 0.00440
80 – 89	0.00796 - 0.01928	0.00486 - 0.01313
90 – 99	0.02108 - 0.03443	0.01469 - 0.02933
100	1.00	1.00

This table contains base-case non-HIV-related mortality for modeled cohorts of PWID. It was calculated using the race/ethnicity-adjusted non-HIV-mortality, and the relative mortality ratios for PWID calculated from NHANES data[25].

NHANES: National Health and Nutrition Examination Survey; **PWID:** people who ever injected drugs; **YNPHIV:** youth with HIV acquired non-perinatally; **YPHIV:** youth with HIV acquired perinatally; **YWoH:** youth without HIV.

eFigure 1.
Projected survival from age 18 years, by sex, and 95% confidence intervals for adult HIV-related mortality inputs

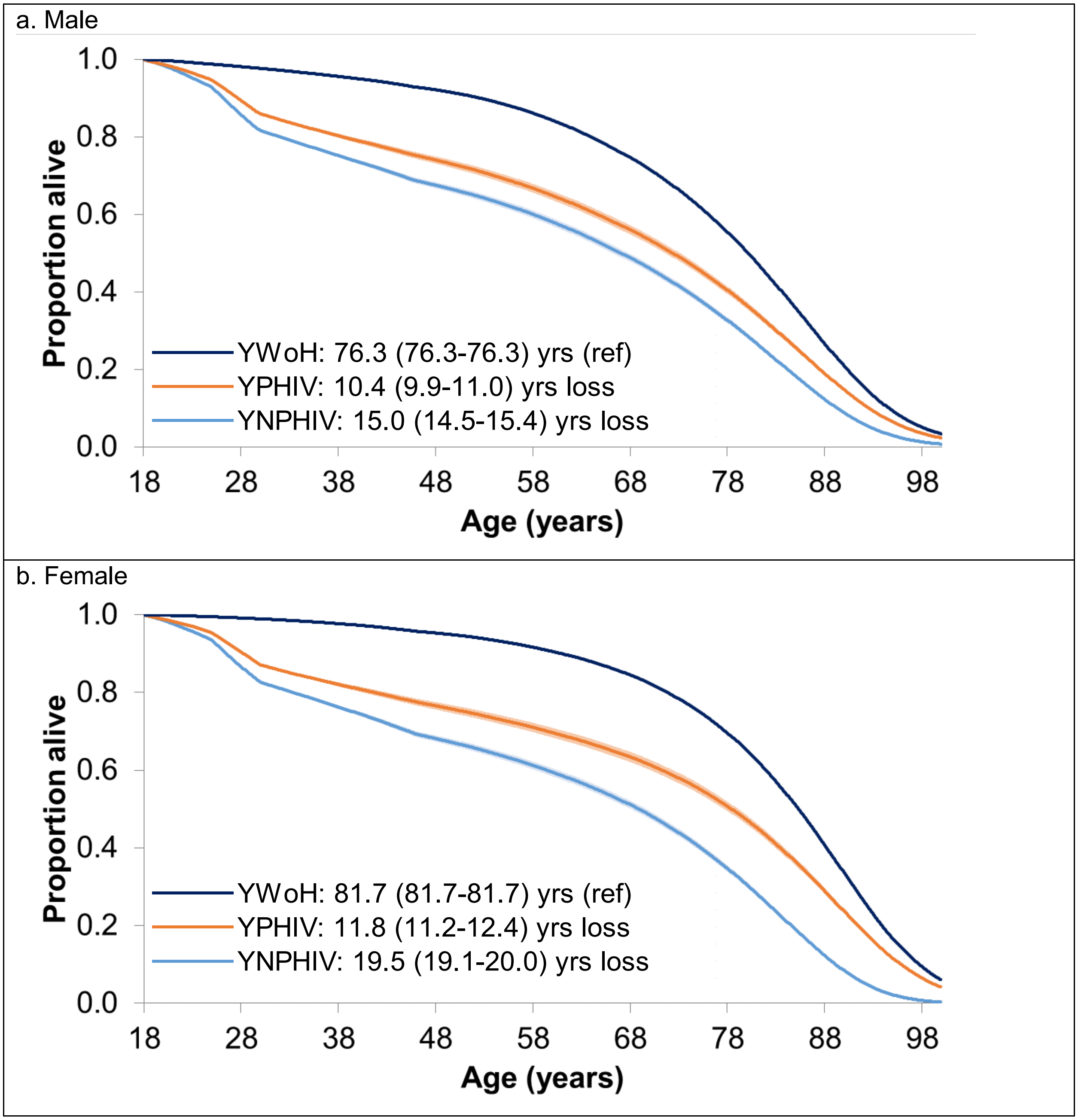

Values in parentheses report the range for sensitivity analyses.
Ref: reference group; **YNPHIV:** youth with HIV acquired non-perinatally; **YPHIV:** youth with HIV acquired perinatally; **Yrs:** years; **YWoH:** youth without HIV.

© 2024 Neilan AM et al. *JAMA Health Forum.*

eFigure 2.
Projected survival from age 18 years, by sex, and risk for HIV acquisition and 95% confidence intervals for relative mortality ratios for HIV acquisition risk

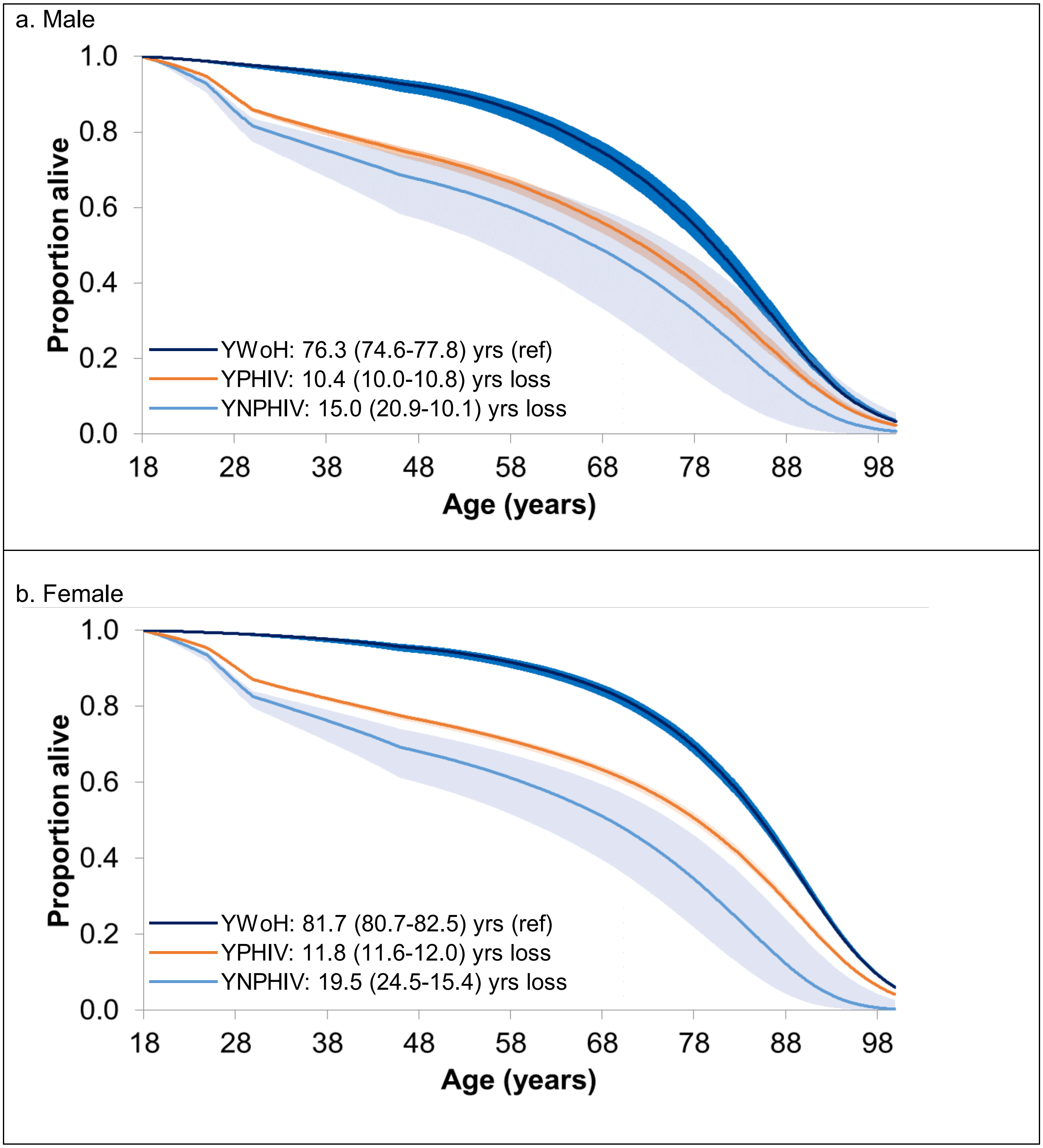

Values in parentheses report the range for sensitivity analyses.
Ref: reference group; **YNPHIV**: youth with HIV acquired non-perinatally; **YPHIV**: youth with HIV acquired perinatally; **Yrs:** years; **YWoH**: youth without HIV.

© 2024 Neilan AM et al. *JAMA Health Forum.*

eFigure 3.
Projected survival from age 18 years, by sex, and sensitivity analysis using non-HIV-related mortality for individuals with average risk for HIV infection in place of non-HIV-related mortality from individuals with income below the federal poverty level

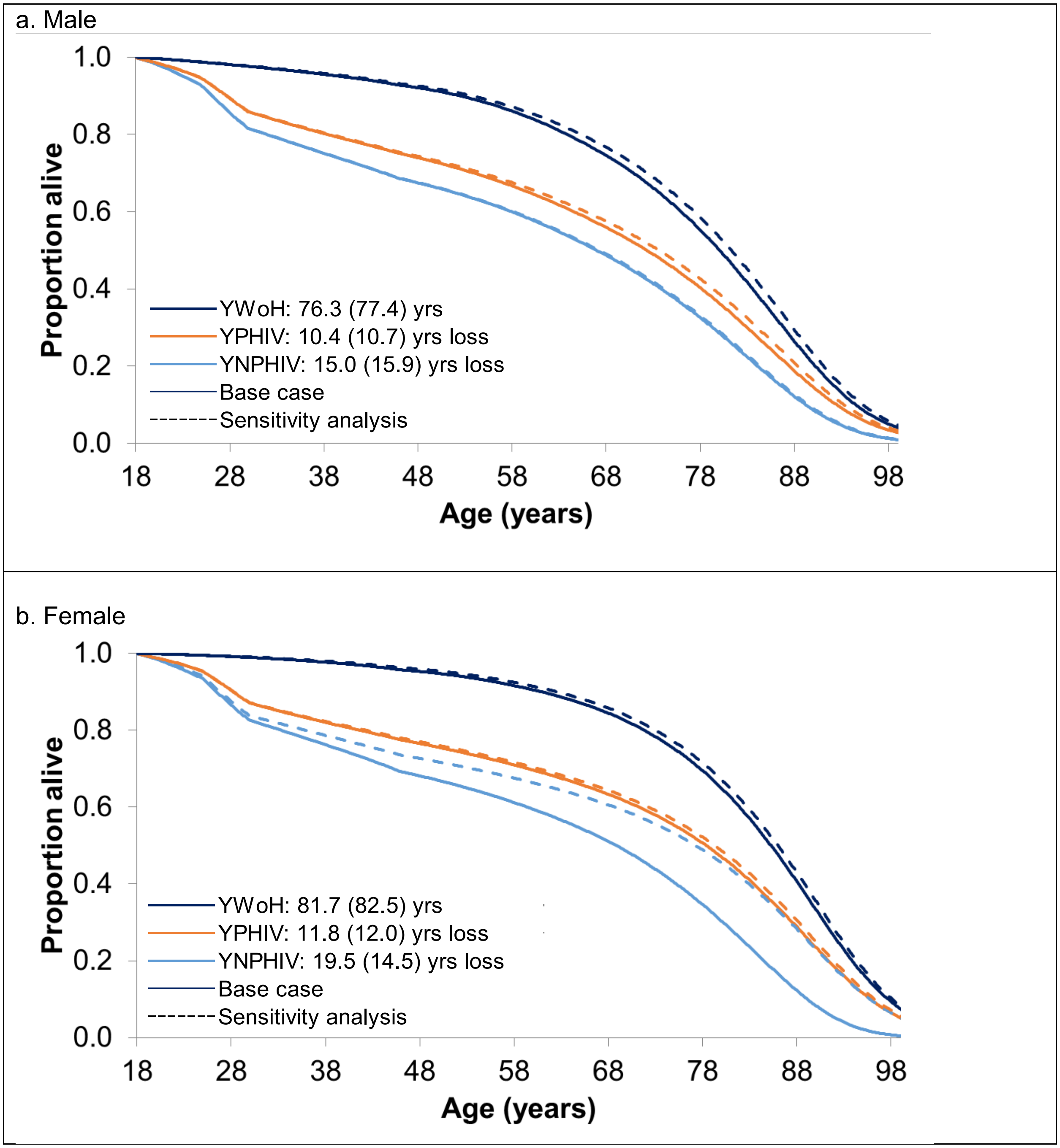

Values in parentheses report the range for sensitivity analyses.
YNPHIV: youth with HIV acquired non-perinatally; **YPHIV**: youth with HIV acquired perinatally; **Yrs:** years; **YWoH**: youth without HIV.

© 2024 Neilan AM et al. *JAMA Health Forum.*

eFigure 4.
Projected survival from age 18 years, by sex, and age cutoffs for relative mortality ratios for HIV acquisition risk

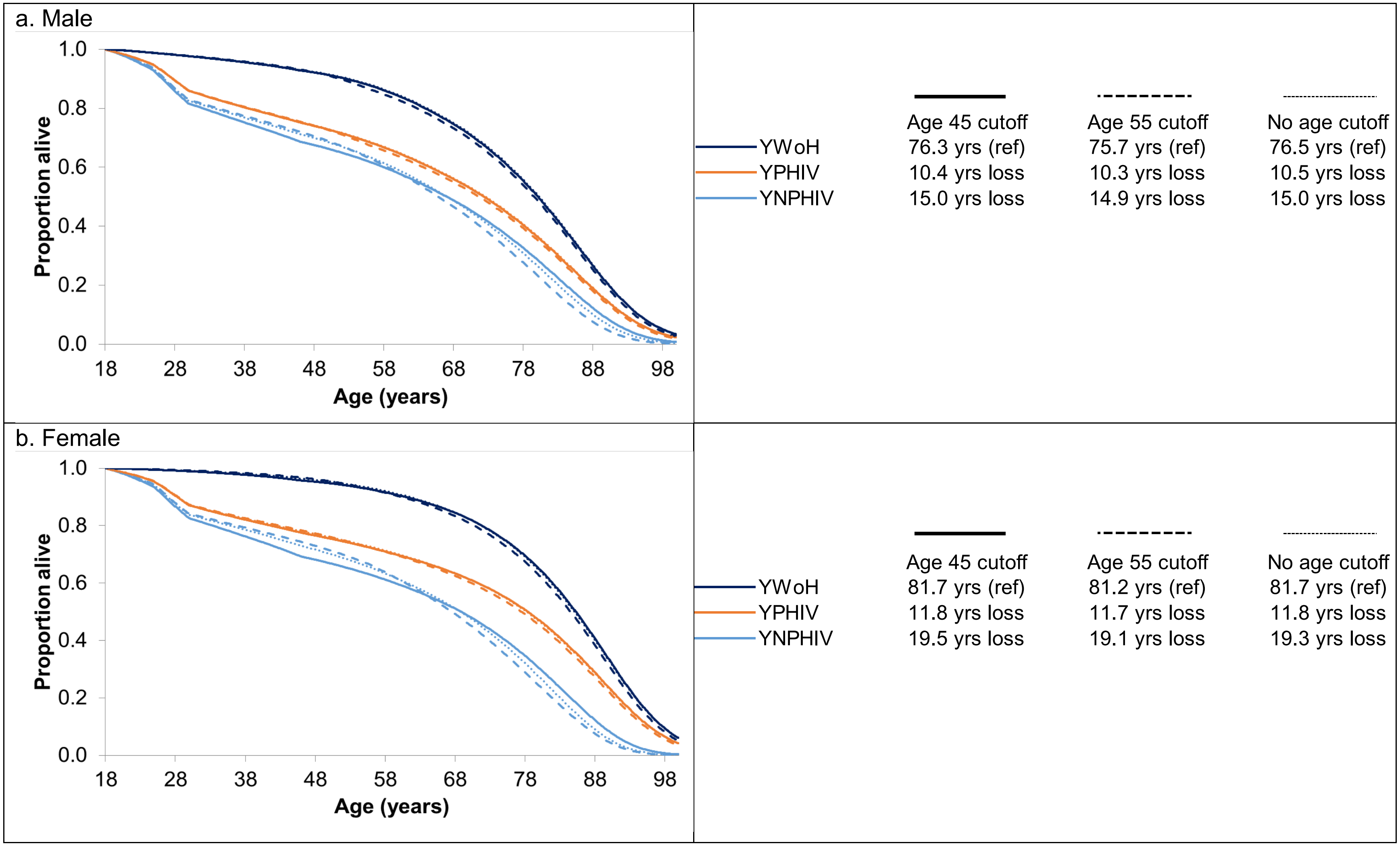

Ref: reference group; **YNPHIV**: youth with HIV acquired non-perinatally; **YPHIV**: youth with HIV acquired perinatally; **Yrs:** years; **YWoH**: youth without HIV.

© 2024 Neilan AM et al. *JAMA Health Forum.*

eREFERENCES

50. Adolescent Medicine Trials Network for HIV/AIDS Interventions | ATN [Internet]. Accessed May 3, 2021. https://atnweb.org/atnweb/

51. Preston SH, Heuveline P, Guillot M. Demography: measuring and modeling population processes. Malden, MA: Blackwell Publishers; 2001.

52. Bureau UC. US population size [Internet]. Census.gov. Accessed April 1, 2021. https://www.census.gov/data/tables/time-series/demo/popest/2010s-national-detail.html

53. Centers for Disease Control and Prevention (CDC). Characteristics associated with HIV infection among heterosexuals in urban areas with high AIDS prevalence --- 24 cities, United States, 2006-2007. MMWR Morb Mortal Wkly Rep 2011;60(31):1045–9. Accessed July 22, 2021. https://www.cdc.gov/mmwr/preview/mmwrhtml/mm6031a1.htm

54. Eddy DM, Hollingworth W, Caro JJ, et al. Model transparency and validation: a report of the ISPOR-SMDM Modeling Good Research Practices Task Force-7. Med Decis Making 2012;32(5):733–43.

55. Centers for Disease Control and Prevention (CDC). National Center for Health Statistics (NCHS). Data Linked to NDI Mortality Files [Internet]. [cited 2021 Jan 11]; Available from: https://www.cdc.gov/nchs/data-linkage/mortality.htm

56. National HIV Behavioral Surveillance (NHBS) | Surveillance Systems | Statistics Center | HIV/AIDS | CDC [Internet]. 2021 [cited 2021 Nov 22]; Available from: https://www.cdc.gov/hiv/statistics/systems/nhbs/index.html

57. Gallagher KM, Sullivan PS, Lansky A, Onorato IM. Behavioral Surveillance Among People at Risk for HIV Infection in the U.S.: The National HIV Behavioral Surveillance System. Public Health Rep 2007;122(Suppl 1):32–8.

58. Ross EL, Weinstein MC, Schackman BR, et al. The Clinical Role and Cost-Effectiveness of Long-Acting Antiretroviral Therapy. Clin Infect Dis 2015;60(7):1102–10.

© 2024 Neilan AM et al. *JAMA Health Forum.*
