# Supplementary material for: Projected Life Expectancy for Adolescents With HIV in the US
Source: JAMA Health Forum. 2024 May 10;5(5):e240816. doi: 10.1001/jamahealthforum.2024.0816 (PMC11087843; doi:10.1001/jamahealthforum.2024.0816)
Supplement: Supplement 2. — Data sharing statement [file jamahealthforum-e240816-s002.pdf]

Data Sharing Statement

Neilan. Projected Life Expectancy for Adolescents With HIV in the US. *JAMA Health Forum*. Published May 10, 2024. doi:10.1001/jamahealthforum.2024.0816

Data
Data available: No

Additional Information
Explanation for why data not available: No data were collected for this study. We are happy to make input parameterization and model-based analyses available for review. Please contact Dr. Anne Neilan, first author, at aneilan@mgh.harvard.edu if data sharing is requested.
